# 23S rRNA modifications stimulate catalytic activity and prevent the formation of alternative structures

**DOI:** 10.1093/nar/gkag800

**Published:** 2026-08-13

**Authors:** Daniel S D Larsson, Aivar Liiv, Rya Ero, Jaanus Remme, Maria Selmer

**Affiliations:** Department of Cell and Molecular Biology, Uppsala University, BMC, P.O. Box 596, SE-75124 Uppsala, Sweden; Institute of Molecular and Cell Biology, Riia 23, University of Tartu, 51010 Tartu, Estonia; Institute of Molecular and Cell Biology, Riia 23, University of Tartu, 51010 Tartu, Estonia; Institute of Molecular and Cell Biology, Riia 23, University of Tartu, 51010 Tartu, Estonia; Department of Cell and Molecular Biology, Uppsala University, BMC, P.O. Box 596, SE-75124 Uppsala, Sweden

## Abstract

Ribosomal RNA (rRNA) modifications cluster around the peptidyl transferase centre (PTC), the catalytic centre of the ribosome, yet their collective functional roles remain unclear. Here we analyse *Escherichia coli* ribosomes lacking 11 or 12 modifications near the PTC. Using kinetic assays, we show these hypo-modified ribosomes catalyse peptide bond formation at rates twofold to threefold lower than wild-type and exhibit reduced thermal stability. Cryo-electron microscopy of hypo-modified ribosomes reveals multiple alternative conformations of the PTC and exit tunnel regions, disrupting native stacking and hydrogen bonding critical for positioning of transfer RNA substrates. These findings indicate that rRNA modifications stabilize the native PTC structure, preventing formation of alternative, nonfunctional conformations and thereby enhancing catalytic efficiency. Our study provides insight into how rRNA modifications fine-tune ribosome function by maintaining structural integrity essential for efficient translation.

## Graphical Abstract

**Graphical Abstract:**
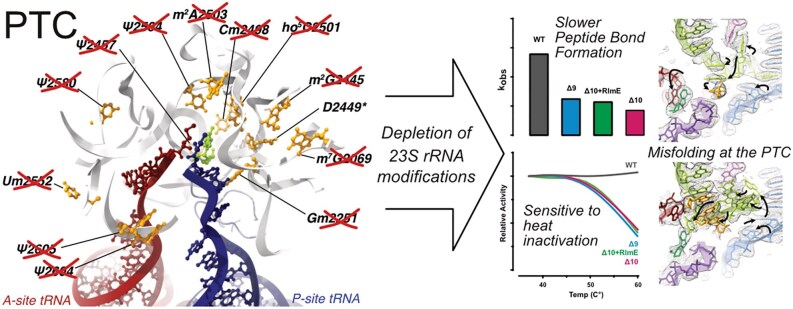

## Introduction

Stable RNA species contain modified nucleotides (MNs) that are often located at conserved sites. In the ribosomal RNA (rRNA) of bacteria, most MNs are located around the decoding centre, the peptidyl-transfer centre (PTC), and the inter-subunit bridges. Methylations and pseudouridylations are the most frequent of these rRNA modifications, which in bacteria are made by site-specific enzymes that act at different stages of ribosome assembly. Although high-resolution structures of ribosomes are available [1–3], the precise contributions of rRNA modification to ribosome structure and function remain largely enigmatic.

**Figure 1.**
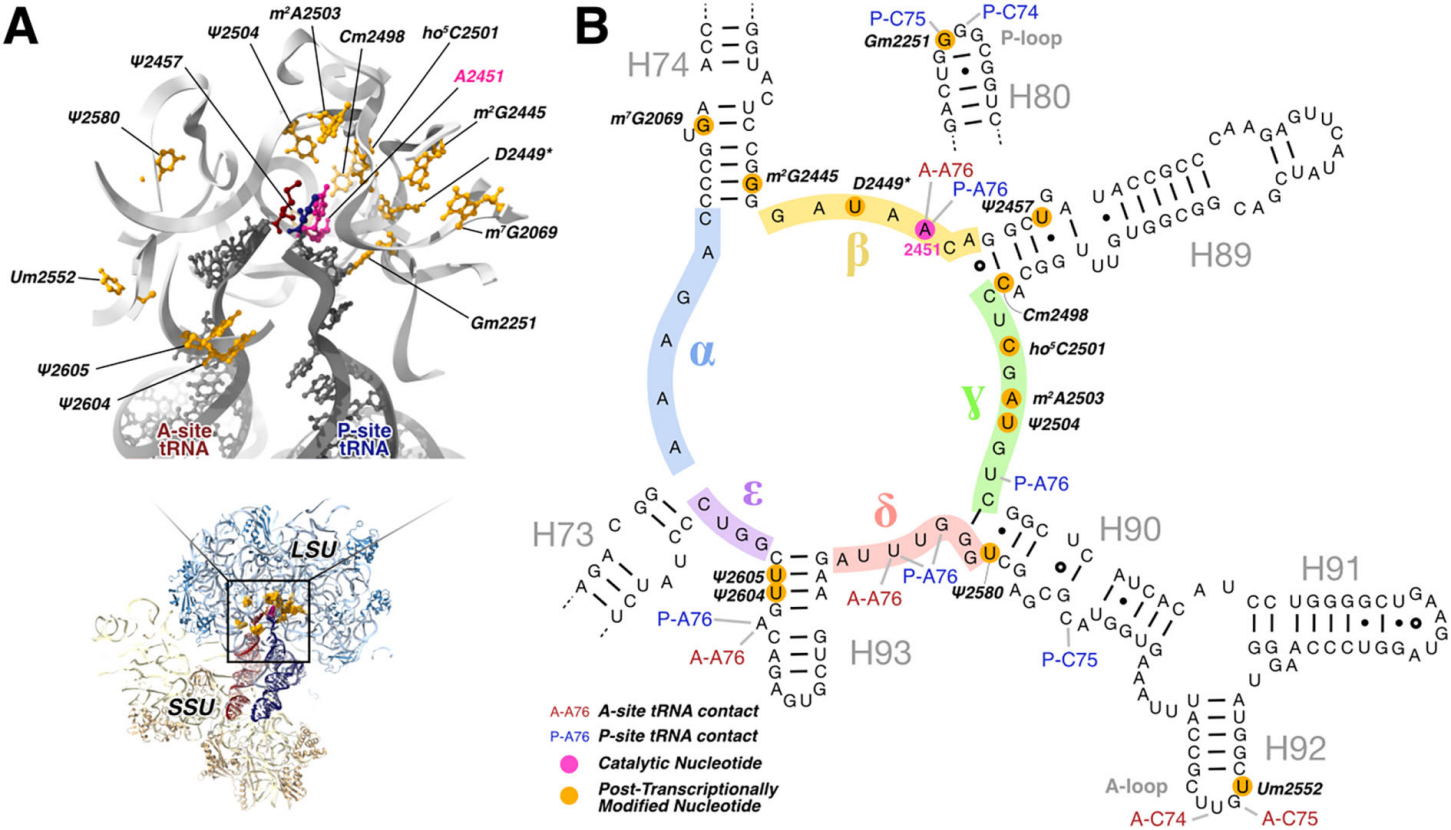
Post-transcriptional modifications at the PTC. (**A**) MNs (orange) within 25 Å of the catalytic residue A2451 (pink). None of the MNs have direct contact to A2451 or to the transfer RNAs (tRNAs). (**B**) Secondary-structure diagram of the PTC region of 23S rRNA domain V. Sections of the PTC ring are labelled with Greek letters α–ε. Nucleotides in contact with tRNA residues in PDB ID 8EMM are indicated. The D2449 modification (*) is present in all ribosomes in this study.

In the *Escherichia coli* ribosomal large subunit (LSU), 13 out of the 36 known MNs [4] are located around the peptidyl transferase centre in domain V of 23S rRNA, within 25 Å of the catalytically essential A2451 residue (Fig. 1). These include five pseudouridins (Ψ2457, Ψ2504, Ψ2580, Ψ2604, and Ψ2605), three 2′O ribose methylations (Gm2251, Cm2498, and Um2552), and three base methylations (m^7^G2069, m^2^G2445, and m^2^A2503), as well as one dihydrouridine (D2449) and one 5-hydroxycytidine (ho^5^C2501) [1]. The genes encoding the corresponding enzymes are all known, but RdsA (D2449), which was only identified recently [5, 6], is not included in this study. Recently, hypoxia-induced methylation at carbon 5′(*S*) of ribose moieties of dihydrouridine at position 2449 (D^5S^m2449) and 2′-*O*-metylcytidine at position 2498 (Cm^5S^m2498) by RlmX was identified [6]. A total of 10 modification enzymes (MEs) make 12 modifications in the domain V and one in the domain II (Ψ955) of 23S rRNA (Supplementary Table S1). Across species, highly conserved modifications around the PTC are Gm2251, Um2552, Ψ2457, and ho^5^C2501. Interestingly, there are also several examples of conserved tertiary interactions in ribosome structures, where stabilizing methylations occur on different nucleotide interaction partners across kingdoms of life [7].

The functions of MNs have primarily been studied using knock out (KO) strains. Single knock-outs of rRNA modification enzyme genes, with the exception of RlmE, which 2′O methylates U2552, do not significantly affect bacterial growth or translation [8, 9]. In contrast, simultaneous depletion of nine or more enzymes leads to severe defects in growth and ribosome assembly [10]. Interestingly, deletion of all seven rRNA-specific pseudouridine synthase genes results in only a weak phenotype in *E. coli* [11]. However, deletion of 10 MEs targeting domain V of 23S rRNA is possible, but the resulting strain shows severe growth retardation, defects in LSU assembly and cold sensitivity [10].

High-resolution structural studies have shown how MNs contribute to stabilizing the native rRNA structure in ribosomes from *E. coli* and other species [1–3]. Generally, pseudouridines tend to form water-mediated hydrogen bonds with phosphate oxygens of the same nucleotide, thereby stabilizing the rRNA backbone [12, 13], while base methylation increases base stacking propensity [14] and ribose methylations influence the ribose conformation [15]. Methylations are frequently found in regions of tertiary interactions.

Two conserved 2′O methylated nucleotides are located at the A (Um2552) and P (Gm2251) loops of 23S rRNA. Deletion of the RlmE gene, which specifically adds the Um2552 methylation results in decreased growth rate and major defects in LSU assembly [16–19]. Although the rate of dipeptide formation between fMet–tRNA and Phe–tRNA is similar on the ΔRlmE and WT (wild-type) ribosomes, the rates of EF-G dependent translocation and ribosome subunit association are reduced in ΔRlmE ribosomes [19]. The strong phenotype caused by RlmE depletion is further aggravated by deletion of the pseudouridine synthase RluC, which modifies positions 955, 2504, and 2580, especially at lower temperatures [20]. In contrast, single deletion of *rlmB* (responsible for Gm2251) does not affect bacterial fitness or ribosome assembly [21].

The clustering of MNs around the PTC argues for their functional importance. Nevertheless, MNs are not strictly required for the peptidyl transferase (PTase) reaction, as *in vitro* transcribed unmodified 23S rRNA of thermophilic bacteria [22, 23] and *E. coli* [24] can catalyse peptide bond formation. However, the rate of the PTase reaction with unmodified 23S rRNA has not been analysed. Despite detailed knowledge of the local interactions of MNs in the mature ribosome, the combined effects of MNs on rRNA folding and ribosome function remain poorly understood. Here, we analyse peptide bond formation on the ribosomes lacking chemical modifications in 23S rRNA around the PTC. Ribosomes lacking 11 or 12 MNs in the domain V of 23S rRNA catalyse peptide bond formation at a significantly reduced rate compared to the WT ribosomes and exhibit an increased entropic penalty of the reaction. MNs confer thermotolerance to ribosomes, indicating stabilization of the native structure. Using single-particle cryogenic electron microscopy (cryo-EM) analysis of programmed 70S ribosomes lacking 12 or 13 MNs, we show that MNs limit the structural heterogeneity of 23S rRNA, particularly its inherent propensity to form alternative stacked conformations. Consequently, ribosomes with a hypomodified PTC region display lower occupancy of A-site tRNA and of tRNA CCA ends correctly positioned for peptidyl transfer.

## Materials and methods

### Ribosome preparation

*Escherichia coli* strains *Δ9, Δ10*, and *Δ10 + RlmE* are described in [10]. The *ΔrlmE* strain was constructed by P1 transduction using *ΔrlmE* of the Keio collection as a donor and *MG1655* as the recipient. Bacteria were cultivated in 2xYT (16 g tryptone, 10 g yeast extract, and 5 g NaCl per 1 l distilled water with pH adjusted to 7.0) or Luria Bertani (LB) media supplemented with relevant antibiotics, and inoculated with a single colony and grown to mid-log phase. Cells were collected by low-speed centrifugation and resuspended in OV-10 lysis buffer [100 mM KCl, 50 mM Tris–HCl, pH 8.0, 6 mM Mg(OAc)_2_, 16% sucrose (w/v), and 6 mM 2-mercaptoethanol] supplemented with DNase I (10 U/ml). Cells were disrupted with glass beads using Bertin Precellys24 Tissue Homogenizer (three cycles of 60 s on/off 6000 rpm at 4°C). Lysate was clarified by centrifugation (13 000 rpm for 15 min at 4°C) and diluted 1:1 with OV-10 lysis dilution buffer [100 mM KCl, 20 mM Tris–HCl, pH 8.0, 10 mM Mg(OAc)_2_, and 6 mM 2-mercaptoethanol]. A total of 80 (A_260_) units of lysate were layered onto a 10%–30% (w/v) sucrose gradient in OV-10 lysis dilution buffer. Ultracentrifugation was carried out (ω^2^t = 3.0 × 10^11^ rad/s) at 4°C using a Beckman Coulter SW-28 rotor. The ribosome profile was determined by continuous monitoring of absorbance at 260 nm. Fractions corresponding to 70S ribosomes and free 50S subunits were collected and stored at −80°C.

The 50S and 30S ribosomal subunits were isolated from 70S ribosomes under dissociating conditions. Briefly, 70S ribosome pellets were resuspended in OV-1 buffer [100 mM KCl, 20 mM Tris–HCl, pH 8.0, 1 mM Mg(OAc)₂, and 6 mM 2-mercaptoethanol]. A total of 80 A₂₆₀ units of 70S ribosomes were layered onto a 10%–25% (w/v) sucrose gradient prepared in the same buffer and subjected to ultracentrifugation at 4°C using a Beckman Coulter SW-28 rotor (ω²t = 2.8 × 10¹¹ rad²/s). Following centrifugation, the 50S and 30S fractions were collected, and the Mg(OAc)₂ concentration was adjusted to 10 mM to stabilize the subunits. The subunits were then pelleted by ultracentrifugation using a Beckman Coulter Ti45 rotor (ω²t = 3.0 × 10¹¹ rad²/s). Finally, the ribosomal subunits were resuspended in polymix buffer [20 mM HEPES–KOH, pH 7.6, 95 mM KCl, 5 mM NH₄Cl, 0.5 mM CaCl₂, 5 mM Mg(OAc)₂, 8 mM putrescine, 1 mM spermidine, and 1 mM dithioerythritol (DTT)].

### Kinetic measurements

All experiments were performed in polymix buffer at 37°C. The kinetics of dipeptide ([³⁵S]fMet-puromycin) and tripeptide (fMet[³H]Phe-puromycin) formation were analysed using a RQF-3 rapid quench-flow apparatus (KinTek Corp., Austin, TX). The 30S initiation complex (30SIC) was assembled by incubating 30S ribosomal subunits (1 μM) with 2.5 μM A52 messenger RNA (mRNA; CAA UUA AGG AGG UAU ACU AUG UUC GUA GCG AGC UAA; Microsynth), 2 μM [³⁵S]fMet–tRNA^fMet^ (6000 dpm/pmol), and initiation factors IF1, IF2, and IF3 (each at a twofold molar excess relative to ribosomes) at 37°C for 15 min.

For the dipeptide assay, pre-formed 70S initiation complexes (70SIC) were assembled by incubating the 30SIC (1 μM) with 0.5 μM 50S subunits at 37°C for 15 min. The complexes were isolated by centrifugation through a 1 ml sucrose cushion (20% w/v sucrose in buffer A) at 260 000 × *g* for 2 h using an Optima MAX-XP ultracentrifuge (Beckman Coulter). The resulting ribosome pellets were resuspended in polymix buffer, shock-frozen in liquid nitrogen, and stored at −80°C.

The rate of [³⁵S]fMet-puromycin formation (dipeptide assay) was determined by rapidly mixing equal volumes of 1 μM 70SIC and preincubated 20 mM puromycin at 37°C for defined time intervals. Reactions were quenched by the addition of 17% (v/v) formic acid. To hydrolyse the tRNA, 150 μl of 6 M KOH was added, followed by neutralization with 500 μl of 1.5 M Tris–HCl (pH 8.8). The reaction products were extracted with 1 ml of ethyl acetate, and [³⁵S]fMet-puromycin was quantified from the organic phase by scintillation counting.

In the tripeptide assay, the rate of peptide bond formation between dipeptidyl–tRNA (fMet[^3^H]Phe–tRNA^Phe^) and puromycin was measured on post-translocation 70S initiation complexes (post70SIC). Briefly, 70SIC and elongation mixture (EM) were formed separately. 70SIC was formed by incubating 30SIC (1 μM) with purified 50S subunits (0.75 μM) at 37°C for 15 min. EM contained 20 μM EF-Tu, 5 μM EF-G, 10 μM tRNA^Phe^, 1 μM [^3^H]phenylalanine ([^3^H]Phe specific activity 5000 dpm/pmol), and Phe–tRNA synthetase, supplemented with 1 mM ATP, 1 mM GTP, 10 mM phosphoenolpyruvate, and 0.05 mg/ml pyruvate kinase for energy regeneration. Post70SIC complex carrying fMet[^3^H]PhetRNA^Phe^ in the P-site was formed by mixing equal volumes of 70SIC and EM, incubated at 37°C for 15 min and isolated by centrifugation through 1 ml sucrose cushions (20% sucrose w/w in buffer A) at 260 000 × *g* for 2 h (Optima MAX-XP, Ultracentrifuge; Beckman Coulter). Ribosome pellets were dissolved in polymix buffer, shock-frozen in liquid nitrogen, and stored at −80°C.

Formation of fMet[^3^H]Phe-Pmn was measured upon rapid mixing of equal volumes (15 μl) of purified pre-translocation 70SIC complex and Pmn (f.c. 20 mM) in the quench-flow apparatus. After the desired incubation time, the reaction was quenched with 17% formic acid, tRNA was hydrolysed by the addition of 150 μl 6M KOH, neutralized by the addition of 500 μl 1 M Tris, pH 8.0, and the tripeptide fMet[^3^H]Phe-Pmn was analysed by extraction with ethyl acetate and subsequent scintillation counting. Note that unreacted [^3^H]Phe remains in the water phase at alkaline pH. Time points (10, 20, 40, 80,…) ms were analysed. The time courses of three independent experiments were fitted to single-exponential kinetics according to the formula: F^fMet-Puro^ = F0*(1 − exp[−k_obs_ * t]), where F^fMet-Puro^ denotes the fraction of the fMet-puromycin formed, F0 is the maximal amount of the puromycin-reactive 70S complexes, and k_obs_ is the apparent first-order rate constant.

### Thermal inactivation assay

Twenty picomoles of 50S ribosomal subunits were incubated for 30 min in OV-10 buffer (100 mM KCl, 20 mM Tris–HCl, pH 8.0, 10 mM Mg(OAc)₂, and 6 mM 2-mercaptoethanol) at various temperatures (37°C, 42°C, 49°C, and 60°C), followed by gradual cooling to 37°C over a 30-min period. PTase activity was assessed using the [³⁵S]fMet-puromycin assay by measuring the reaction product at the 2.5-min time point. Briefly, 15 pmol of 30S subunits were preincubated with initiation factors, A52 mRNA, and [³⁵S]fMet–tRNA^fMet^ for 20 min at 37°C to form the 30SIC. Subsequently, 10 pmol of the thermally treated 50S subunits were added. The reaction was initiated by the addition of puromycin (f.c. 10 mM), and the formation of [³⁵S]fMet-puromycin was measured after 2.5 min.

### Cryo-EM sample preparation

50S subunits were isolated from 70S ribosomes of *Δ9, Δ10* and *ΔrlmE* strains as described above. 50S subunits (final concentration 1 µM) from deletion strains (Δ9, Δ10, and ΔRlmE) were assembled with wild-type 30S subunits (2 µM) into 70S ribosomes in the presence of uncharged tRNA^fMet^ (4 µM), tRNA^Phe^ (4 µM), and synthetic mRNA (8 µM) containing a Shine–Dalgarno sequence followed by an initiator codon and a phenylalanine codon (5′-caauuaaggagguauacuAUGUUCAAAGUACGAUCAAUCU ACGUAUAAUAAAAGAAAAGAAAAGAAAAGGACAUCA CAGAUUAAcgcacaaaaaaaaaaaaaaaaaaaaa-3′). The assembly reactions were carried out in RS-6 Buffer (20 mM Tris–HCl, pH 7.5; 100 mM NH₄Cl; 6 mM magnesium acetate; 1 mM DTT) in a total volume of 150 µl at 37°C for 20 min. 70S ribosomes were purified by 10%–25% sucrose gradient centrifugation in RS-6 Buffer using an SW28 rotor (Beckman Coulter; *w²t* = 2.7 × 10¹¹). The 70S-containing fractions were pooled, and the magnesium acetate was adjusted to 10 mM, and sucrose was removed by dilution and concentration using a 100 kDa molecular weight cut-off Amicon Ultra centrifugal filter unit (Millipore). The final ribosome storage buffer (RS Buffer) consisted of 2 mM Tris–HCl (pH 7.5), 100 mM NH₄Cl, 10 mM magnesium acetate, and 1 mM DTT. To ensure high tRNA occupancy, 1 µM of each uncharged tRNA was freshly added to the sample before freezing.

The samples were incubated for 30 s on QuantiFoil R 2/2 300 mesh copper grids coated with a continuous layer of 2-nm amorphous carbon before blotting for 4 s and plunge frozen in liquid ethane using a VitroBot mark IV (Thermo Fisher Scientific, Waltham, MA, USA) kept at 4°C and 95% humidity. The grids were activated using a EasiGlow (TedPella Inc, Redding, CA, USA) set to 20 mA for 20 s in 0.4 mBar residual air.

### Cryo-EM data collection

Grids were screened on a Glacios (Thermo Fisher Scientific) microscope. The Δ9 and Δ10 data sets were collected on a Titan Krios G3 (Thermo Fisher Scientific) equipped with a K2 (Gatan, Pleasanton, CA, USA) camera at 105 000× magnification (equivalent to a calibrated pixel size of 0.8215 Å/pixel in the sample plane using) with a BioQuantum (Gatan) energy filter with a slit width of 20 eV and with a total dose of 30.0 e/Å^2^ saved as 20 frames. The ΔRlmE data set was collected on a Titan Krios G3 equipped with a K3 camera (Gatan) and BioQuantum energy filter at a nominal magnification of 105 000× (calibrated to 0.8228 Å/pixel) and an energy filter slit width of 20 eV and with a total dose of 40.6 e/Å^2^ saved as 39 frames.

### Cryo-EM data processing

Data were processed using Relion [25] and CryoSPARC [26] according to the conventional single-particle processing pipeline. In short, the movies were motion corrected using the Relion 3 [27] implementation of the MotionCor2 algorithm [28] with 5 × 5 patches. The initial CTF parameters were estimated using Gctf [29], and particles were picked by matching round (Laplacian of Gaussian) templates to features in the micrographs. Particles were extracted, binned 4 × 4, and several rounds of 2D classification were used to remove nonribosomal particles. A consensus 3D reconstruction of the 70S ribosome was obtained after iterative rounds of 3D reconstruction and estimation of optical parameters using the Relion 4 algorithm for per-particle defocus and higher order aberrations [25], and particle polishing [27]. Focused classification without alignment into six classes within a soft-edged mask over the tRNA binding sites, based on the consensus 70S reconstruction after subtracting the signal of the LSU and the small subunit (SSU) segregated particles into classes with tRNA density in the A, P, and/or E sites. Relevant APE, AP, PE, and E classes were reconstructed. The occupancy of tRNA in each class was estimated based on the local loss of contrast in the reconstructions using Occupy [30]. The polished particles from the consensus reconstruction were imported into CryoSPARC 4 and were 3D classified into 10 classes without alignment based on a 40-Å spherical soft-edged mask over the PTC region after subtracting the signal of the LSU and the SSU. Maps were globally sharpened using a negative B-factor based on fitting to the Guinier plot and low-pass filtered based on the estimated Fourier shell correlation (FSC) resolution. The ΔRlmE dataset was processed in the same way, but entirely in CryoSPARC 4, using the Patch Motion for motion correction, the Patch CTF algorithm for initial CTF estimation, and the blob picker for particle picking. The magnification for each dataset was calibrated by rigid-body docking PDB ID 7K00 [1] in the consensus maps in ChimeraX [31] and maximizing the map-model correlation by changing the voxel size.

### Model building and figure preparation

Atomic structures were generated based on PDB IDs 8CF1, 8CGJ, 8CGK [32], and 7K00 [1]. The chains were rigid-body docked into the consensus ΔRlmE structure using ChimeraX and Coot [33] version 0.9.8.95. Residues outside of density or with validation issues were interactively refined or rebuilt using Coot. The tRNA^Phe^ in the A-site, uL5 and 23S helices H25, H42–45, and H74–76 were modeled based on AlphaFold 3 [34] predictions. The E-site tRNA was modeled as tRNA^fMet^, although it is occupied by a mixture of tRNA^fMet^ and tRNA^Phe^. The consensus Δ9 and Δ10 structures were modelled based on the refined consensus ΔRlmE model. Atomic models for residues close to the PTC-region (45-Å distance from A2451) were modeled in the maps for ΔRlmE, Δ9, and Δ10 classes from the focused classification of the PTC-region. The final structures were refined using Servalcat [35] version 0.4.77 with jelly-body restraints. The CCP4 monomer library [36] was used to restrain bond lengths, angles, torsions, planarity, and chirality for standard residues, while restraints for modified residues were generated by Grade2 using the Grade Web Server (https://grade.globalphasing.org, Global Phasing Ltd, Cambridge, UK). The models were validated using Coot, Phenix [37] version 1.21, and MolProbity [38] as distributed in the Phenix package [37]. Refinement and validation information is summarized in Supplementary Table S5. All structure figures were made with ChimeraX [31].

### Statistical analyses

Statistical analysis of experimental data were done using the ordinary two-way analysis of variance (ANOVA) tests and multiple comparison was done using uncorrected Fisher’s least significant difference (LSD) tests.

## Results

### Pre-steady-state kinetics of peptide bond synthesis on ribosomes

We set out to analyse the importance of 23S rRNA modifications during peptide bond formation on the ribosome. To this end, ribosomes were isolated from *E. coli* strains lacking either 11 (*Δ9*) or 12 (*Δ10*) modifications around the PTC, which show accumulation of incompletely assembled ribosomes [10]. Since the aim was to analyse the PTase activity that supports viability of the strains, we isolated fully assembled 70S ribosomes, performed subunit dissociation in low Mg^2+^ to isolate the hypomodified 50S subunits, and combined these with WT 30S subunits in all experiments. To determine the kinetic parameters of peptide bond synthesis, we employed an assay system utilizing puromycin (Pmn) as acceptor substrate and peptidyl–tRNA analogs (fMet–tRNA in the dipeptide assay and fMetPhe–tRNA in the tripeptide assay) as the donor substrate. We selected the puromycin reaction as a model system for peptide bond synthesis, as this system does not involve the multistep accommodation of aa–tRNA to the ribosomal A site [39]. In this assay, 70S complexes (70S*fMet–tRNA*mRNA or 70S*fMetPhe–tRNA*mRNA) were formed in the presence of purified translation initiation and elongation factors, purified via sedimentation through a sucrose cushion and kept at −80°C until use. The same assay system was previously used to define the effect of mutations in both 23S rRNA and ribosomal protein L27 on the rate of peptide bond formation [40, 41].

**Figure 2.**
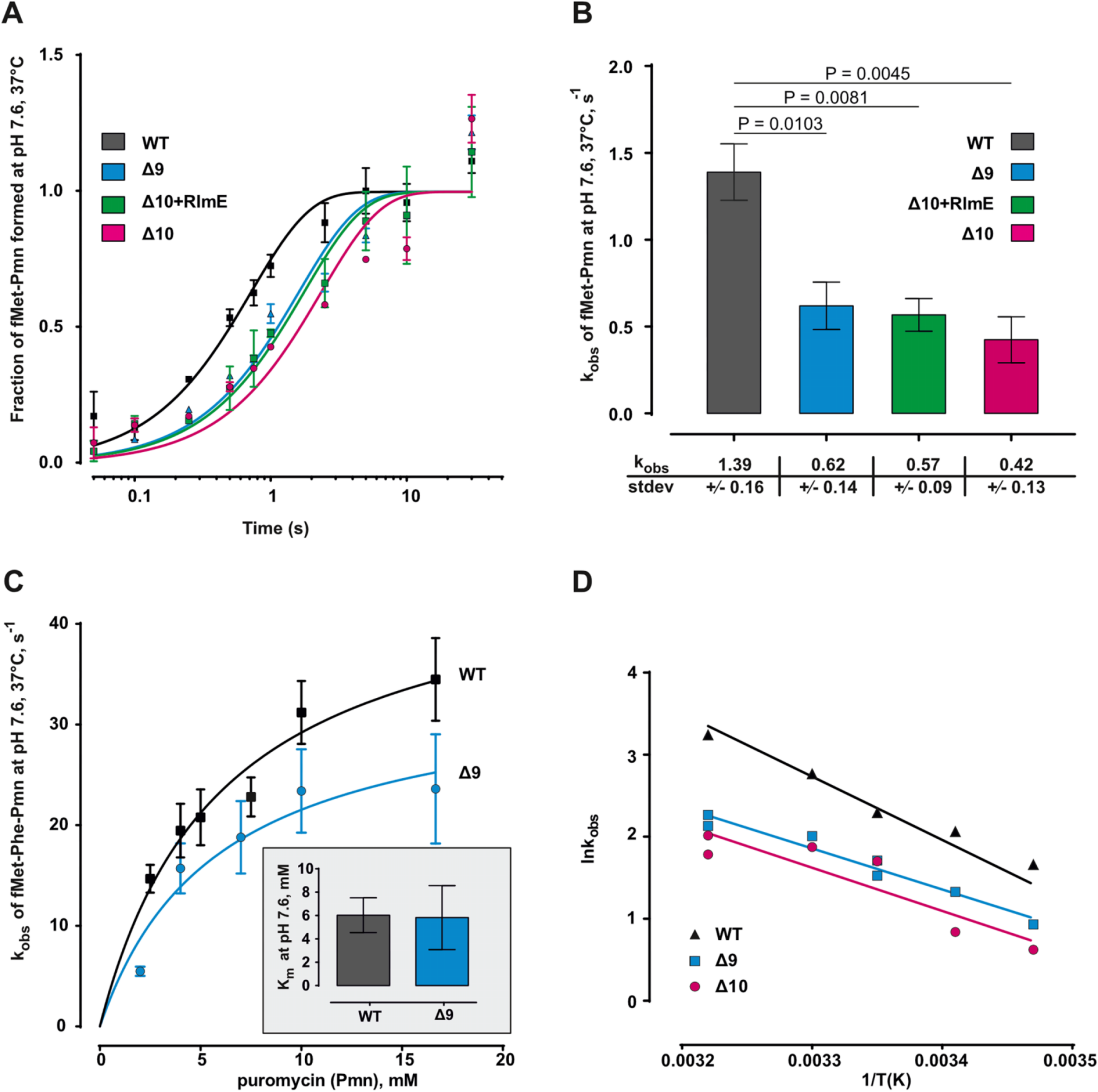
Modifications in the domain V of 23S rRNA stimulate peptide bond formation in the fMet–tRNA and puromycin reaction (dipeptide assay) and in the fMetPhe–tRNA and Pmn reaction (tripeptide assay). Ribosomal 50S subunits were isolated from 70S ribosomes of WT, *Δ9, Δ10*, and *Δ10* + *RlmE* strains. In dipeptide assay fMet–tRNA*mRNA*70S ribosome complex (70SIC) was mixed with an equal volume of 20 mM Pmn in a quench-flow machine and incubated for 0.1–10 s. In tripeptide assay of fMetPhe–tRNA*mRNA*70S complex was reacted with Pmn. (**A**) Time course of dipeptide PTase reaction. **(B**) k_obs_ values of PTase reaction with various ribosomes (dipeptide assay). (**C**) Titration of fMetPhe–tRNA*mRNA*70S complex with Pmn at pH 7.2. Inlet K_M_ values of the PTase reaction on the ribosomes of WT and *Δ9* strain. (**D**) Effect of temperature (15–37°C) on the PTase reaction using tripeptide assay shown as Arrhenius plot.

In the dipeptide assay, the rate constant k_obs_ for the PTase reaction on WT ribosomes was 1.39 ± 0.13 s^−1^ (Fig. 2A and B) at 37°C, in agreement with earlier measurements [41, 42]. Ribosomes of *Δ9* and *Δ10* strains catalysed dipeptide formation with rates of 0.62 ± 0.14 and 0.42 ± 0.13 s^−1^, respectively (Fig. 2A and B). 50S subunits of the *Δ10* strain expressing plasmid-encoded RlmE (*Δ10 + RlmE*), containing the same 23S rRNA modification pattern as Δ9 ribosomes, exhibited k_obs_ of 0.57 ± 0.09 (Fig. 2A and B). Thus, hypo-modified ribosomes catalyse peptide bond synthesis 2.2–3.3 times slower than fully modified WT ribosomes, and RlmE appears to stimulate the PTase reaction slightly. We conclude that some of the 23S rRNA modifications around the PTC stimulate the rate of the PTase reaction.

The PTase reaction rates between fMet–tRNA and Pmn on WT, Δ9, and Δ10 ribosomes were measured at pH 7.2 and 7.6 (Supplementary Fig. S1). The PTase reaction was about three times faster on WT ribosomes than on Δ10 ribosomes at both pH values. Ribosomes of the *Δ9* strain exhibited slightly higher activity than the Δ10 ribosomes (Supplementary Fig. S1). The reaction rate constant (k_obs_) increased 3.7-fold at the higher pH for all three ribosome variants (Supplementary Fig. S1), consistent with previously published data with puromycin as acceptor substrate [39]. Thus, the lack of modifications in domain V of 23S rRNA does not appear to affect the pH dependence of the PTase reaction.

Peptide bond synthesis between fMet–tRNA and Pmn is slow as compared to the translation elongation rate *in vivo* (1.4 versus 20 s^−1^) (Fig. 2B) [39]. However, with dipeptidyl–tRNA (fMetPhe–tRNA) as donor substrate, the PTase reaction rate is more than an order of magnitude higher [39, 40, 43]. Therefore, the tripeptide assay was used to determine the effect of 23S rRNA modification on the rate of PTase reaction. Indeed, the maximal rate of peptidyl-Pmn synthesis was about 35 s^−1^ on the WT ribosomes at pH 7.6 and saturating Pmn concentration (Fig. 2C). In agreement with the earlier observations [39, 40, 43], this is about 25 times faster than with fMet–tRNA as a donor substrate, likely explained by additional hydrogen bonds between dipeptidyl–tRNA and 23S rRNA that stabilize the active PTC conformation [44]. To test whether the puromycin affinity to the ribosomal A-site is affected by the absence of modifications, the tripeptide assay was performed at various Pmn concentrations on WT and Δ9 ribosomes (Fig. 2C). For this experiment, ribosomes from the *Δ9* strain were used, as in this strain, ribosome assembly is not significantly affected at 37°C [10], but as described above, ribosomes from *Δ9* and *Δ10* showed similar rates in the dipeptide assay. For WT and Δ9 ribosomes, the K_M_ value was 6 mM for both WT and Δ9 ribosomes (Fig. 2C). Thus, the PTC modifications do not affect the affinity of puromycin to the ribosomal A site.

Temperature dependence (15–37°C) of peptide bond formation on the ribosomes from the WT, *Δ9*, and *Δ10* strains was analysed using the tripeptide assay. This assay was done at pH 7.2 to improve the solubility of puromycin at low temperatures. Both Δ9 and Δ10 ribosomes catalyse the PTase reaction at a similar rate, slower than the rate on WT ribosomes at all temperatures (Supplementary Table S2). However, the difference in k_obs_ values between the WT and hypo-modified ribosomes increases with increasing temperature. Thus, the functional significance of the 23S rRNA modifications in peptide bond formation appears stronger at higher temperatures. At 37°C, the WT ribosomes catalyse peptide bond formation about three times faster than hypo-modified ribosomes, similar to the results of the dipeptide assay. These results suggest that 23S rRNA modifications around the PTC are important for stabilizing the binding of either substrate or a reaction intermediate to the ribosome.

The first order rate constants from the tripeptide assay were used to calculate the thermodynamic parameters of the PTase reaction from an Arrhenius plot (Fig. 2D and Table 1). The values of parameters ΔH and TΔS of the reaction of WT ribosomes were very similar to the previously observed values [45]. Comparison of WT and hypo-modified ribosomes demonstrates that the ribosomes of *Δ9* and *Δ10* strains exhibit similar values for all thermodynamic parameters but deviate significantly from WT ribosomes. Surprisingly, the activation energy (Ea) and enthalpy (ΔH) are higher on the WT ribosomes than on the Δ9 and Δ10 ribosomes, suggesting that modifications around the PTC increase the activation energy barrier of the reaction. On the other hand, the TΔS value of the PTase reaction is significantly higher on the Δ9 and Δ10 ribosomes. Thus, the modifications around the PTC reduce the entropic penalty of peptide bond formation on the ribosome. Reduced enthalpy and increased entropy can result from a weakened binding of substrates to the PTC. This idea is consistent with the hypothesis that one or more rRNA modifications near the PTC are crucial for substrate orientation during peptidyl transfer. Alternatively, rRNA modifications can stabilize the binding of the reaction intermediate to the PTC.

**Table 1.** Thermodynamic parameters of the fMetPhe–tRNA reaction with Pmn (tripeptide assay) on the ribosomes of WT and hypo-modified strains *Δ9* and *Δ10*

	E_a_ kcal/mol	Standard deviation (SD)	ΔH kcal/mol	SD	ΔG kcal/mol	SD	TΔS kcal/mol	SD
WT	15.53	±1.72	14.94	±0.62	16.09	±0.08	1.16	±0.69
Δ9	10.94	±1.01	10.35	±1.01	16.44	±0.05	6.10	±1.05
Δ10	10.59	±2.43	10.00	±2.43	16.45	±0.1	6.45	±2.53

### Modifications stabilize the 50S subunit structure

**Figure 3.**
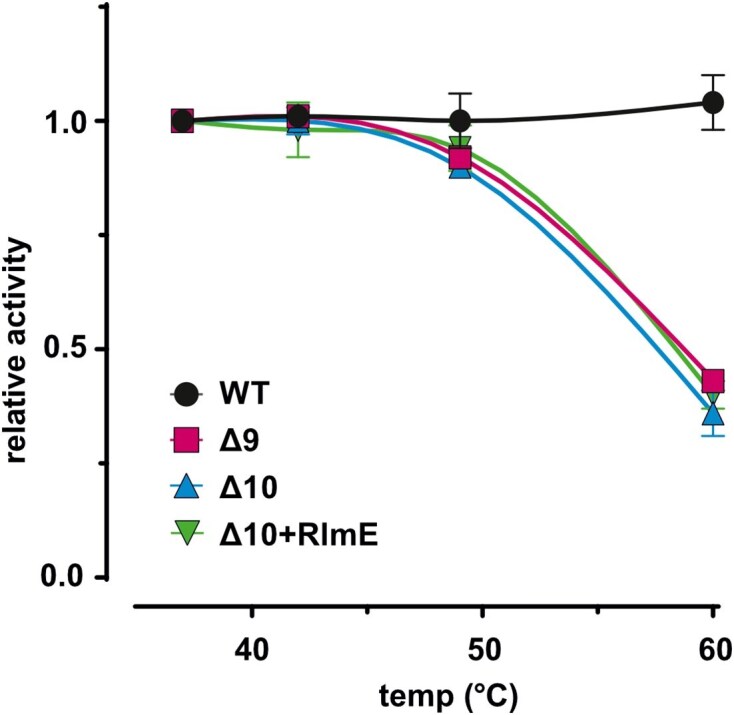
Effect of 23S rRNA modifications on the stability of 50S subunits. Ribosomal 50S subunits were preincubated at various temperatures, and 70SIC was formed with fMet–tRNA, and the active fraction of ribosomes according to PTase activity was determined using Pmn as an acceptor substrate.

The lacking rRNA modifications around the PTC appears to affect the rate of peptide bond formation more at higher temperatures (Supplementary Table S2 and Fig. 2D), suggesting a possible problem with ribosome stability. If rRNA modifications stabilize the ribosome structure, a lack of modifications is expected to cause reduced thermotolerance. To test this, the 50S subunits were first pre-incubated for 30 min at 37°C, 42°C, 49°C, or 60°C, followed by measurement of PTase activity. Pre-incubation of the WT 50S subunits at any temperature did not affect their ability to catalyse peptide bond formation (Fig. 3), in agreement with [46]. In contrast, pre-incubation of the Δ10 50S at 49°C reduced PTase activity by 10%, and at 60°C by 60% (Fig. 3). The 50S subunits of *Δ9* and *Δ10* + *RlmE* strains exhibited similar stability at elevated temperatures as Δ10, albeit the reduction of PTase activity was slightly lower as compared to the Δ10 ribosomes (Fig. 3). These results demonstrate that the modifications around the PTC are important for LSU stability. Although RlmE is important for 50S assembly [16–19], its resulting methylation, Um2552, has little impact on the LSU stability, as evidenced by similar thermotolerance of the 50S subunits of *Δ9* and *Δ10* strains. The results demonstrate that the 23S rRNA modifications around the PTC protect ribosomes from heat inactivation and are important for ribosome structural stability. However, since heat inactivation of hypo-modified 50S subunits occurs during prolonged incubation at 49°C or higher temperatures but not at 37°C, it does not explain the reduced PTase activity of the Δ9 and Δ10 ribosomes at 30°C and 37°C.

### Structure determination of hypomodified ribosomes by cryo-EM

To shed light on the structural consequences of depleting the 70S ribosome of its native PTC-region modifications, we employed single-particle cryo-EM analysis. Grids were prepared with Δ9, Δ10, and, as a control, ΔRlmE 70S ribosomes programmed with a short synthetic mRNA, deacylated tRNA^fMet^ at the P-site, and deacylated tRNA^Phe^ at the A-site. Cryo-EM analysis yielded consensus 70S ribosome reconstructions at better than 1.9 Å resolution for all three samples (Supplementary Figs S2–S7).

The structure of the ΔRlmE 70S ribosome is overall very similar to the WT *E. coli* 70S ribosome [1, 32]. In contrast, the Δ9 70S structure, in which the RlmE modification Um2552 is present, showed regions of weak density at the PTC, resulting from flexibility and partial misfolding of 23S rRNA as well as r-protein uL4. Alternative 23S rRNA conformations were mainly observed in the PTC ring (regions α–ε, as defined in Fig. 1B and Supplementary Table S3). In particular, nucleotides 2058–2062 (in PTCα), 2447–2453 (in PTCβ), and 2501–2506 (in PTCγ) showed significant heterogeneity with evidence of multiple conformations. The Δ10 70S ribosome showed additional disorder and misfolding, including also nucleotides 2581–2585 (in PTCδ) and the A-loop (in H92). Consequently, we observed non-native conformations of the CCA end of the A-site tRNA in all three samples (see below).

The presence or absence of methylations was confirmed directly in the maps at all nucleotide positions that were ordered in the consensus reconstructions (Supplementary Fig. S8). As a consequence of lacking methylation, several unmodified amine or hydroxyl groups were observed to coordinate structured water molecules (G2069, C2498, and U2552) or to form noncanonical polar contacts (G2251 with D2449, discussed below). The nonplanar base geometry of D2449 was resolved in the ΔRlmE and Δ9 maps and the posttranscriptionally added hydroxyl group of ho^5^C2501 was visible in ΔRlmE (Supplementary Fig. S8). The two isomers pseudouridine and uridine are isosteric and thus cannot be distinguished by shape in the maps, but we observed an increased distance from C5 of uridine to the closest structured water in U2457, U2580, and U2605 (Supplementary Fig. S8).

Focused 3D classification on the tRNA binding pockets of the three reconstructions segregated the particles into classes depending on their tRNA occupancy in the A, P, and E sites (Supplementary Table S4). Particles with A-site tRNA were fewer in Δ9 ribosomes compared to ΔRlmE and accounted for only 26% in Δ10. Even after classification, the tRNAs showed weaker density than the ribosome. Thus, tRNA occupancy was estimated based on local loss in contrast to be 45%, 35%, and 21% in the A site, and 70%, 61%, and 45% in the P site of the ΔRlmE, Δ9, and Δ10 ribosomes, respectively (Supplementary Fig. S9).

Diffuse density around the PTC indicated structural heterogeneity, and motivated further focused classification. Using a spherical mask around the PTC, it was possible to resolve multiple alternative conformations, in particular for Δ9. Notably, the PTC classes with WT-like conformation also showed high occupancy of A-site tRNA.

### Misfolding of the PTC ring

**Figure 4.**
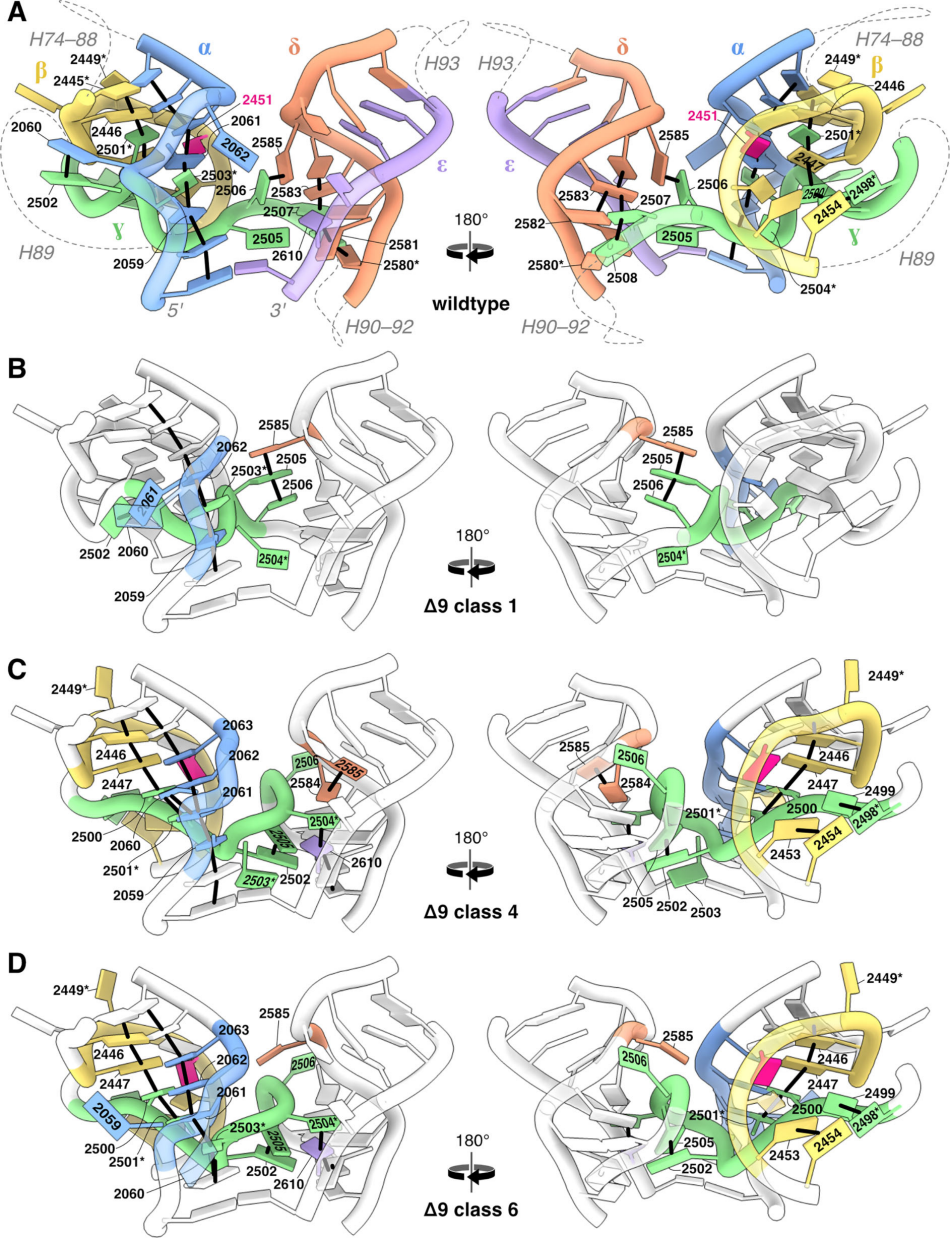
Misfolding of the PTC ring in 70S Δ9 ribosomes with A- and P-site tRNA. PTCα, PTCβ, PTCγ, PTCδ and PTCε are coloured as in Fig. 1B. In panels (B)–(D), only nucleotides with structural changes compared to WT are coloured. Black bars highlight selected base stacks. Supplementary Figs S11–S13 show detailed views of PTCα and PTCδ for all PTC classes. (**A**) WT conformation (PDB 8EMM). Dashed lines indicate helices connected to the PTC ring. (**B**) Δ9 class 1. Detailed views in Figs 5B and D. (**C**) Δ9 class 4. Detailed views in Fig. 5F and H. (**D**) Δ9 class 6. Detailed views in Fig. 5J and L.

**Figure 5.**
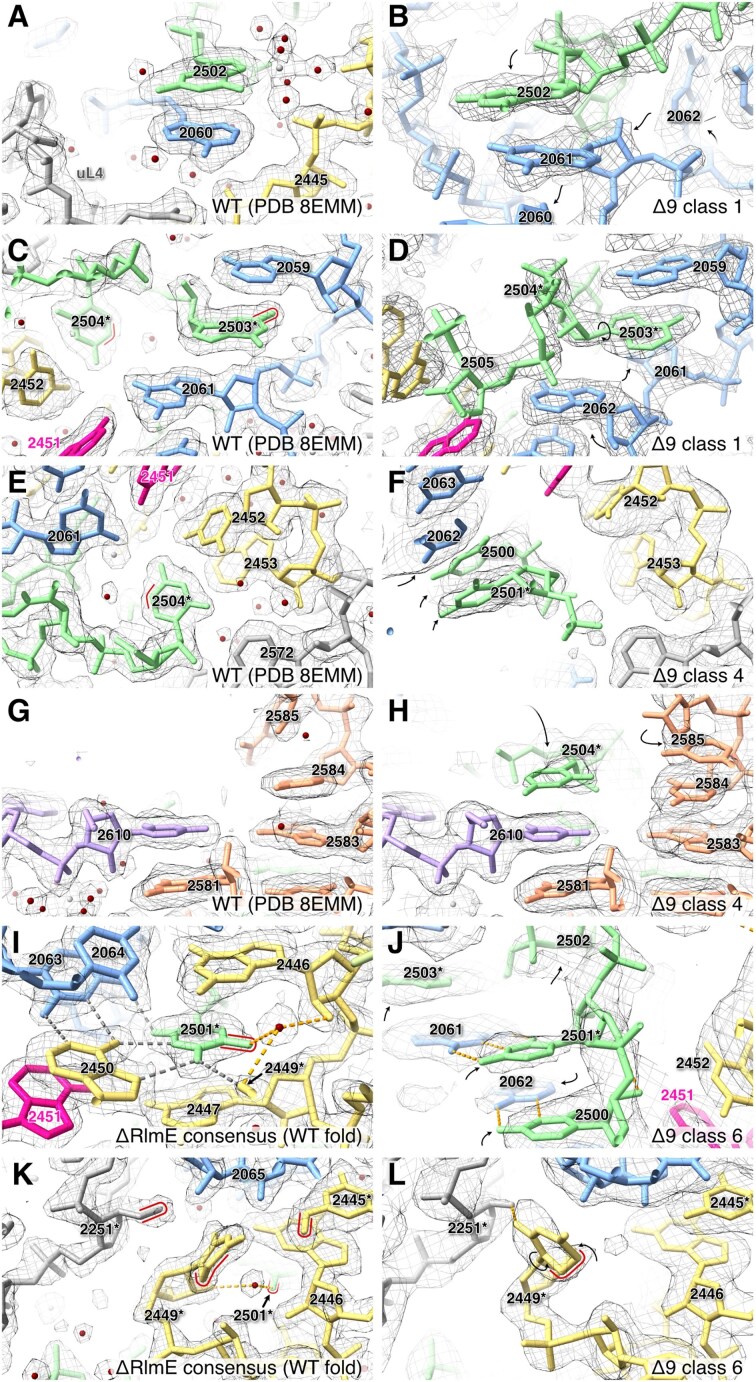
Comparison of local interactions in native and misfolded PTC-ring structures. Colours as in Fig. 1B. Water molecules are shown as red spheres and inorganic ions as white spheres. Dashed lines indicate polar contacts. Red outline indicates modification. *Asterisk indicate MN in WT. (**A**) G2502 stacks with A2060 in WT ribosomes. (**B**) In Δ9 class 1, G2502 is repositioned and stacks with G2061, which has been displaced by A2062. (**C**) m^2^A2503 stacks with A2060 in WT ribosomes. (**D**) In Δ9 class 1, A2503 (unmodified) has changed from syn to anti conformation and A2062 displaced G2061. U2504 (unmodified) and G2505 have moved from their WT positions. (**E**) WT conformation of Ψ2504. (**F**) In Δ9 class 4, U2504 (unmodified) has been displaced by U2500 and C2501 (unmodified). (**G**) WT conformation of C2610. (**H**) In Δ9 class 4, U2504 (unmodified) is stacked on top of C2610, and U2585 is stacked on U2584. (**I**) ΔRlmE shows WT fold for ho^5^C2501. (**J**) In Δ9 class 6, C2501 (unmodified) is base-pairing with G2061. (**K**) ΔRlmE shows WT conformation of D2449 with the base sandwiched between the Gm2251 and m^2^G2445 methylation sites. (**L**) In Δ9 class 6, the D2449 base is rotated and forms a hydrogen bond to the unmethylated 2′ hydroxyl group of G2251.

The PTC ring of the 23S rRNA secondary structure (Fig. 1B) folds into a compact tertiary structure, where PTCγ threads through the middle (Fig. 4A). In the native structure, the three MNs of PTCγ (ho^5^C2501, m^2^A2503, and Ψ2504) form an intricate network of tertiary contacts with PTCα, PTCβ, and PTCδ (Supplementary Fig. S10A). In the absence of these modifications, several of the modelled conformations exhibited substantial refolding of PTCγ, characterized by disrupted contacts and a range of connected conformational changes and alternative tertiary interactions (Fig. 4B–D, and Supplementary Figs S10B and S11–S13). Most such interactions are observed in several of the models, of which we highlight some selected examples. In the native structure, G2502 base-stacks with A2060 (Figs 4A and 5A), but in Δ9 class 1 it instead stacks with G2061 (Figs 4B and 5B). The space where G2061 natively stacks with m^2^A2503 (Fig. 5C), is then occupied by A2062 (Fig. 5D). In Δ9 class 4, the WT position of Ψ2504 (Fig. 5E) is replaced by U2500 and (unmodified) C2501 (Fig. 5F), and U2504 instead stacks on top of C2610 (Figs 4C, and 5G and H).

In WT ribosomes, ho^5^C2501 stacks between G2446 and G2447 (Figs 4A and 5I) and forms a base triplet with A2450 and C2063 (Fig. 5I). In addition, the hydroxyl modification makes water-mediated contacts to riboses of G2446 and D2449 (Fig. 5I, orange), which are lost in the hypo-modified ribosomes. In Δ9 class 6, the unmodified C2501 forms a base-pair with G2061, stacked with A2062 (Figs 4C and 5J).

In absence of the stabilizing interaction with ho^5^C2501, the base of D2449 is shifted in most hypo-modified ribosomes (Supplementary Figs S14 and S15). In the most prevalent alternative conformation, the base of D2449 adopts a rotated conformation, enabling hydrogen bonding to the nonmodified ribose hydroxyl group of G2251 (Fig. 5K and L). In WT ribosomes, this contact is prevented by the Gm2251 methylation.

Interestingly, also ΔRlmE ribosomes, which contain all PTC-ring MNs and otherwise display a WT-like fold, include one class (class 2, Supplementary Fig. S11) with refolded PTCα and partially disordered PTCγ, suggesting that WT ribosomes may likewise sample some alternative conformations.

In several Δ9 and Δ10 classes, the misfolded PTCα clashes with the native conformation of the 55–73 loop of r-protein uL4, for which weak density supports an alternative conformation that partially blocks the nascent peptide exit tunnel (Supplementary Fig. S16). Importantly, this uL4 loop forms a restriction point in the exit tunnel of WT ribosomes, and its deletion was shown to induce cold sensitivity, cause accumulation of assembly intermediates, and alter the sensitivity pattern to exit-tunnel binding antibiotics [47].

### Misfolding of the A loop affects binding of A-site tRNA

**Figure 6.**
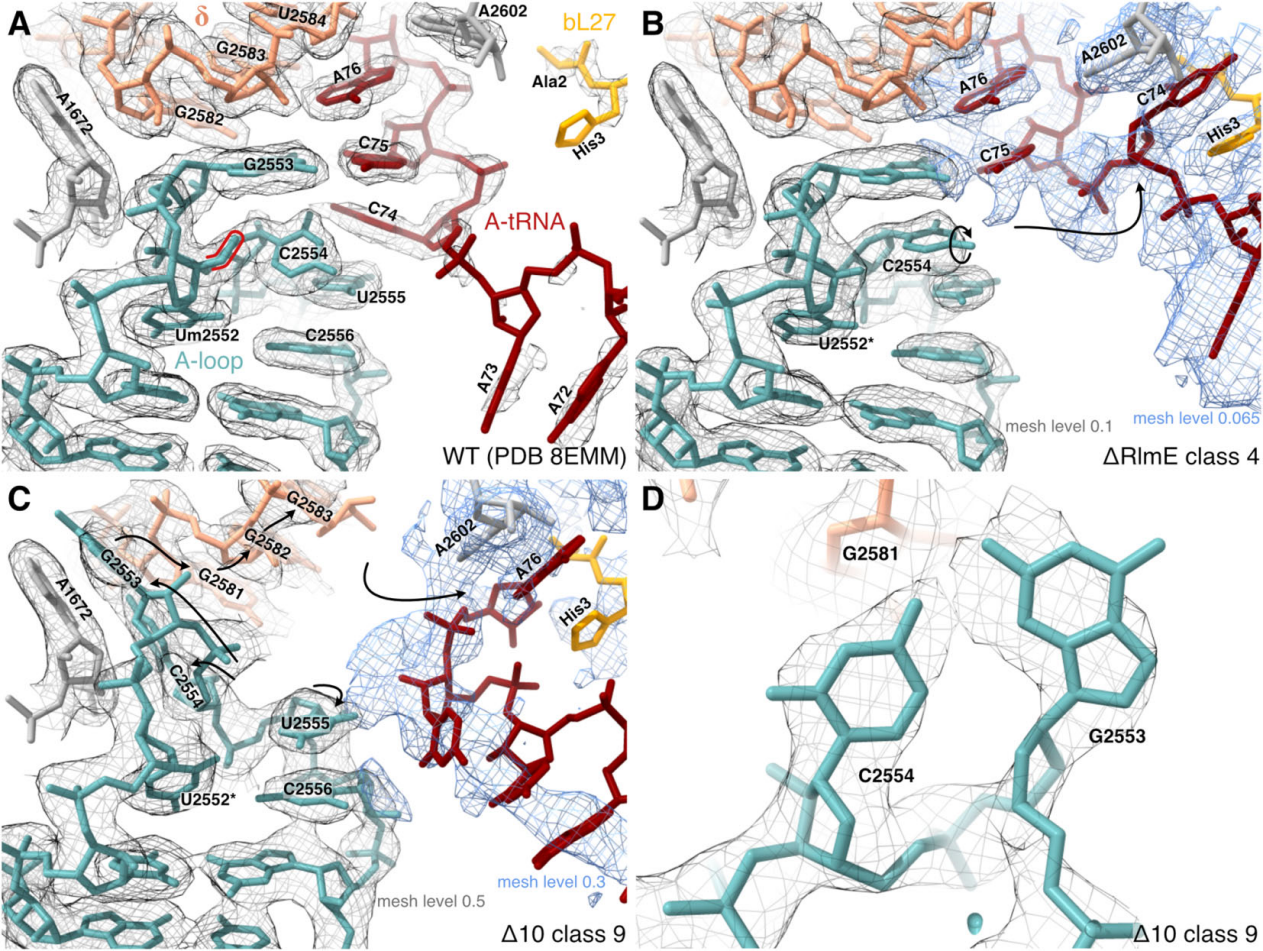
Alternative structures of the A-loop and the A-site tRNA CCA end. The A-loop is shown in blue (modification of Um2552 marked in red), A-site tRNA in dark red, bL27 in orange, PTCδ in salmon, U2506 in mint green, A2602 and A1672 in grey. Segmented maps are shown as grey mesh within 2 Å of residues (blue within 3 Å). Supplementary Figs S17–19 show the same view for all PTC classes. (**A**) Wild-type conformation (PDB ID 8EMM). (**B**) ΔRlmE class 4 with C2554 rotated. C74 of the A-site tRNA is sequestered between A2602 and His3 of bL27. tRNA density shown at lower threshold as blue mesh. *U2552 unmodified. (**C**) Δ10 class 9 with flipped-out G2553 and U2554 stacking between A1672 and G2581, which distorts PTCδ. The CCA end is displaced (tRNA modelled similar to Δ9 class 1). (**D**) Close-up of of G2553 and U2554 in panel (C).

Structures of WT ribosomes with a tRNA in the A site [48, 49] normally show C74, C75, and A76 stacked with each other and C75 base paired to G2553 of the A loop irrespective of if the tRNA carries an amino acid [48] (Fig. 6A) or is deacylated [49]. Here, classes from all three ribosome samples showed examples of alternative conformations of the CCA end of the A-site tRNA (Fig. 6B and C and Supplementary Figs S17–S19) or a vacant A site. In the Δ10 ribosomes, the omission of A-loop 2′O methylation of U2552 in the Δ9 background leads to misfolding of the A-loop (Fig. 6 and Supplementary Fig. S19). In the predominant conformation, G2553 and U2554 are flipped out and form a *cis* Hoogsteen-sugar base pair (Fig. 6C and D). This conformational change explains the lower occupancy of A-site tRNA in Δ10, since G2553 and U2554 cannot form their normal hydrogen bonds to C74 and C75 of the A-site tRNA. The flipped-out bases clash with PTCδ, which is refolded. As a consequence, many polar contacts between the A-loop and PTCδ are disrupted and PTCδ is disordered in most Δ10 classes, affecting e.g. G2583 and its contact with A76 of the A-site tRNA. A similar conformational change of G2553 and U2554 was previously observed in ΔRlmE 50S ribosomes [19] (Supplementary Fig. S17). Programmed 70S ΔRlmE ribosomes on the other hand showed only a minor difference from the WT conformation, but in some classes, the base of U2554 is rotated and fully stacked between the neighbouring bases, clashing with C74 of the A-site tRNA, which is shifted to stack between His3 of bL27 and A2602 (Fig. 6B and Supplementary Fig. S17).

### Alternative stacking of the PTC perturbs the binding site of the A- and P-site tRNA acceptor ends

**Figure 7.**
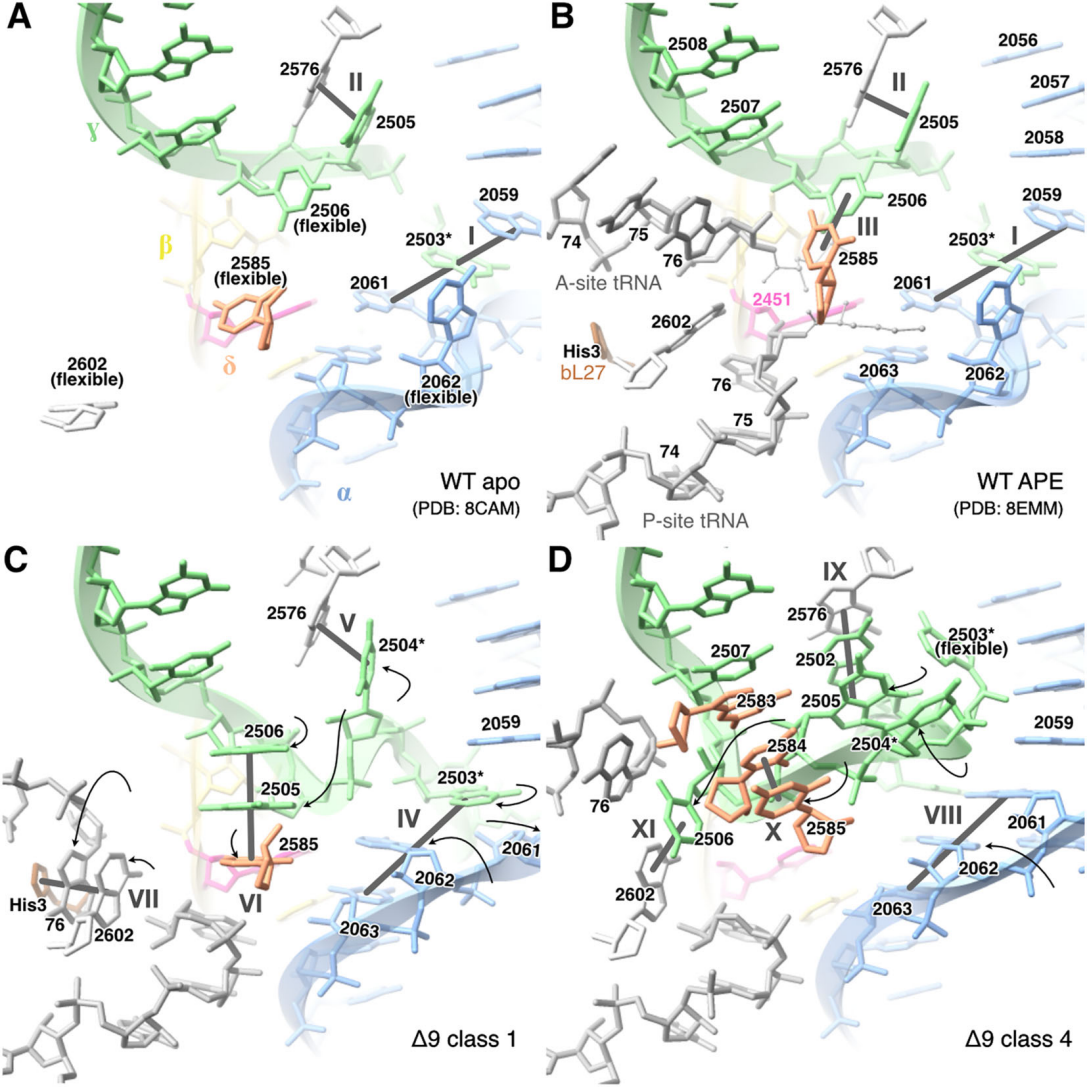
Alternative stacking at the PTC. Residues coloured as in Fig. 1B. Bars indicate WT or misfolded base stacks with roman numbers as in Supplementary Fig. S10B. *Asterisk indicate MN in WT. Supplementary Fig. S20 shows the same panels with maps. Supplementary Figs S21–S24 show all PTC classes. (**A**) WT conformation with no bound tRNA (PDB 8CAM). (**B**) WT conformation in presence of A-site and P-site tRNA (PDB 8EMM). (**C**) Δ9 class 1. (**D**) Δ9 class 4.

In the WT ribosome, U2506, U2585, and A2602 are dynamic in the absence of tRNA (Fig. 7A and Supplementary Fig. S20), but during translation adopt distinct conformations to position the aminoacyl and peptide groups of the tRNAs for peptidyl transfer [50, 51] (Fig. 7B). In the hypomodified ribosomes, these nucleotides adopt several alternative conformations (Fig. 7C and D, and Supplementary Figs S21–S23). In Δ9 class 1, G2505 intercalates between U2506 and U2585 (Fig. 7C) and A2602 together with His3 (bL27) trap A76 of the A-site tRNA in a stack. In Δ9 class 4, U2585 flips around to stack on U2584 while U2506 instead forms a stacking interaction with A2602, creating a barrier between the A76 nucleotides of the A- and P-site tRNAs (Fig. 7D). These misfolded conformations would sterically prevent peptidyl transfer.

### Modifications modulate the conformation of H89

**Figure 8.**
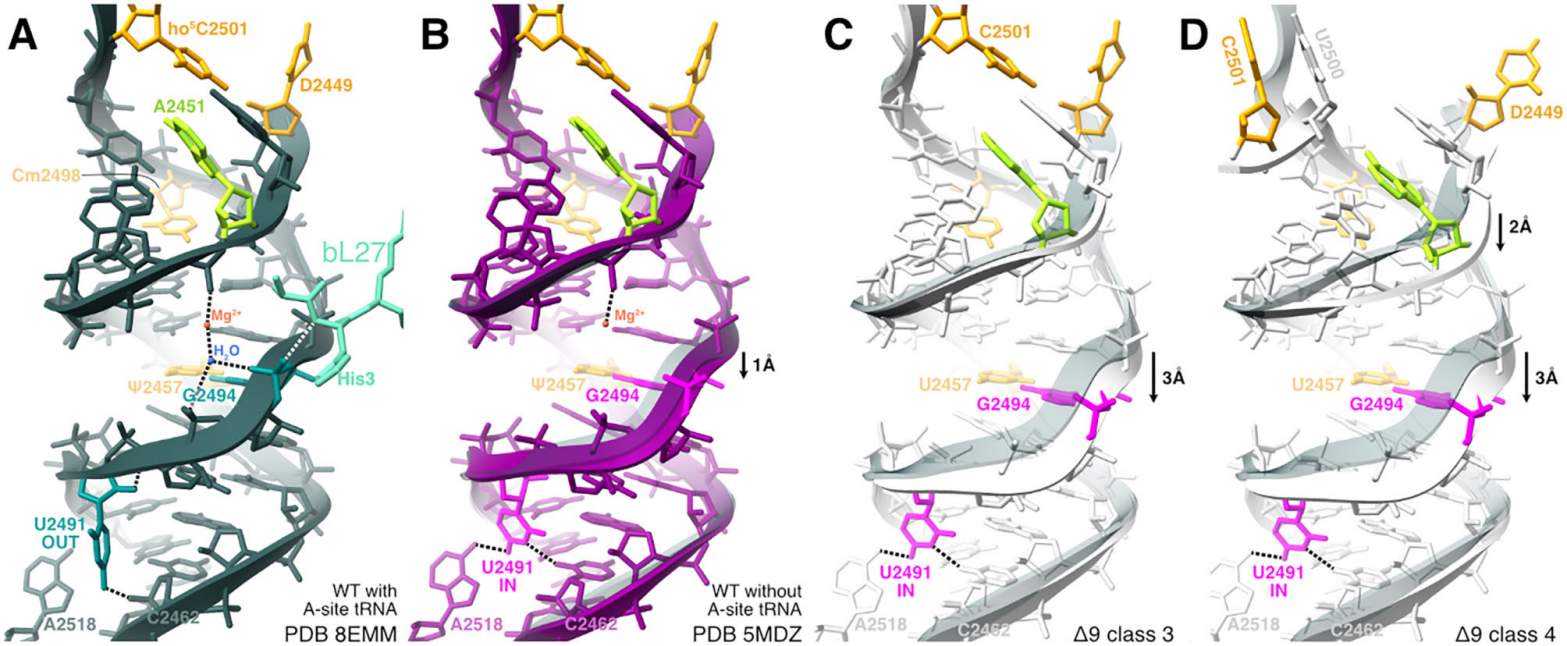
Switching of U2491 and deformation of H89. Supplementary Figs S24–S26 show all PTC classes. (**A**) In WT ribosomes with A-site tRNA (PDB 8EMM), U2491 (teal) adopts an outward conformation (OUT) and hydrogen bonds (dashed lines) to the ribose of C2462. G2494 (teal) has a solvent-bridged contact to A2451 (lime green) bridged by solvent and a hydrogen bond stabilizes the N-terminus of bL27 (mint green). MNs are shown in orange. (**B**) In WT ribosomes with empty A site (PDB 5MDZ), U2491 (magenta) is tucked in and hydrogen bonds to the base of C2462 and to A2518 (IN). The helix is shifted by ∼1 Å at G2494 relative to PDB 8EMM (semi-transparent). (**C**) Δ9 class 3 with empty A site overlayed with PDB 8EMM (semi-transparent). (**D**) Δ9 class 4 with misfolded PTC-ring and empty A site. Catalytic nucleotide A2451 is displaced by ∼2 Å relative to PDB 8EMM (semi-transparent).

Helix H89 has two modification sites (Ψ2457 and Cm2498) and is located between PTCβ and PTCγ and close to the catalytic nucleotide A2451 (Figs 1B and 8A). In most hypomodified ribosome classes, one side of the helix is compressed by up to 3 Å (Fig. 8C and D, and Supplementary Figs S25 and  S26). The shift is correlated with a conformational switching of the unpaired U2491. The switching of this nucleotide has not been previously described, but comparison of WT structures with or without A-site tRNA [44, 52] shows that the U2491 position is correlated with a small compression of the helix in absence of tRNA (Fig. 8A and B). Only the tRNA-bound conformation allows the phosphate of G2494, which base pairs with Ψ2457, to form a polar contact to His3 of bL27, and a magnesium-solvent bridge to the ribose of the catalytically essential A2451. Mutations of U2491 have been shown to affect PTase activity [53]. Only the Δ9 and Δ10 classes with WT conformation of C2501 (unmodified) and D2449 lack the prominent deformation of H89 (Δ9 classes 1, 2, and 7 and Δ10 class 3, Supplementary Figs S25 and S26). This suggests that the MNs at and near the base of H89 serve to maintain the rigidity of the helix, preventing large deformation upon U2491-induced switching, and assuring the correct geometry of the PTC.

## Discussion

Bacteria harbor genes for numerous enzymes dedicated to specific post-transcriptional rRNA modifications, many of which cluster around the PTC. Based on our previous work showing that *E. coli* strains lacking nine or ten genes encoding PTC-region modification enzymes suffer severe growth defects and accumulate 50S assembly intermediates [10], we set out to characterize kinetics and structures of the subset of 50S subunits that are incorporated into Δ9 and Δ10 70S ribosomes and sustain translation in these cells. Thus, as a strategy to understand the collective function of PTC-region rRNA modifications in WT ribosomes, we elucidate the combined effects of their deletion on PTC structure and catalytic activity.

Starting from large single-particle cryo-EM data sets, we were able to resolve an ensemble of misfolded PTC structures in the hypomodified ribosomes. This was technically possible because most of the ribosomal particle remains well-ordered, guiding particle alignment and enabling high-resolution consensus reconstructions. The structural heterogeneity was limited to a well-defined region around the PTC at the centre of the particle, enabling accurate classification of individual particles into sufficiently populated states that guided focused classification. The resulting structural snapshots likely capture a mixture of long-lived and dynamically exchanging states. Building on this, we propose that the same approach could be applied to investigate whether minor populations of alternative PTC conformations are also present in WT translating ribosomes.

Both types of hypo-modified ribosomes catalyse peptide bond formation at a rate threefold lower than that of WT ribosomes (Figs 2 and 3). The determined thermodynamic parameters of the PTase reaction reveal that hypo-modified ribosomes exhibit lower activation energy but substantially higher entropic penalty than WT ribosomes. Together with their temperature sensitivity (Fig. 3), this suggests that hypo-modified ribosomes adopt structures that are less efficient for catalysis. This is supported by the structures of Δ9 and Δ10 ribosomes that show substantial misfolding of the PTC (Figs 4, 7, and 8), with the latter displaying the greatest structural heterogeneity. Still, the purification of 70S ribosomes assured that the sample was not contaminated with assembly intermediates that accumulate in the *ΔrlmE*, *Δ9*, and *Δ10* strains [10, 19].

During the translation cycle, three universally conserved nucleotides U2506, U2585, and A2602 undergo conformational changes to accommodate binding of aminoacyl tRNA or release factors in the A site [50, 51, 54]. Our structures suggest that the stability of the surrounding PTC is fine-tuned to allow localized but not global flexibility of this complex tertiary RNA structure. In particular three MNs in PTCγ seem essential to maintain the native PTC structure. ho^5^C2501, m^2^A2503, and Ψ2504 form a mesh of native tertiary interactions (Figs 4A and 5, and Supplementary Fig. S10A). When they in the hypo-modified ribosomes adopt non-native conformations, this can instead result in wide-spread misfolding of the surrounding structure, to a large degree stabilized by alternative stacking interactions (Figs 4 and 7, and Supplementary Fig. S10B). While all three nucleotide positions are conserved in bacteria, the respective modifications are only present in some lineages [55–57] and the presence of ho^5^C2501 is regulated in relation to hypoxia [6]. This highlights that different species have evolved alternative solutions to the same problem [7]. The mild phenotypes of individual knockouts of these enzymes [8] point to the redundancy in the system and collaborative contributions of the modifications to stabilize the native structure. We can thus draw a parallel between the observed effects of removal of individual or multiple rRNA modifications and the epistatic effects between different mutations in enzyme evolution [58]. In positive epistasis, individual mutations have weak phenotypes, while the combination of mutations has a more dramatic effect than what would be expected from the effects of the individual mutations, similar to observations in relation to rRNA hypomodification.

The only two universally conserved LSU rRNA methylations reside in the A- and P-loops. In the P-loop, the RlmB-mediated 2′O methylation of G2251 blocks formation of a hydrogen bond to a misfolded conformation of D2449 (Fig. 5L). The RlmE-catalysed 2′O methylation of U2552 instead stabilizes the native A-loop conformation through hydrophobic interactions with the surrounding bases. In its absence, we observed unfolding of the A-loop (Fig. 6E), disabling G2553 and U2554 from binding to C75 and C74 of tRNA. Despite the major effects of the *rlmE* knockout in the Δ9 background on structure and growth rate [10], Δ9 and Δ10 ribosomes show very similar PTase activity in the puromycin reaction. This is not surprising, since puromycin in the fragment reaction is expected to interact mainly with G2583 rather than the A-loop, and not require induced fit [50].

Our structures highlight the inherent propensity of large RNAs to adopt alternative stacked conformations. Much like a molecular game of musical chairs, the alternative stacking of one base often displaces another, triggering conformational shifts that propagate across numerous nucleotides (Figs 4 and 7). We cannot exclude that some of these alternative conformations may occur at low frequency also in WT ribosomes. Evolution has introduced redundancy in rRNA modifications, resulting in the weak phenotypes observed in single knockouts, and where each modification subtly influences the equilibrium between the native structure and a spectrum of potential misfolded states.

## Supplementary Material

gkag800_Supplemental_File

## Data Availability

The cryo-EM maps and models in this study have been deposited in the Electron Microscopy Data Bank (https://www.ebi.ac.uk/emdb/) under the accession codes EMDB-55349, 55427–55430, 55432–55437, 55415, 55467–55476, 55618, 55702–55711 and in the Protein Data Bank under the DOIs https://doi.org/10.2210/pdb9SYH/pdb, https://doi.org/10.2210/pdb9T19/pdb, https://doi.org/10.2210/pdb9T1A/pdb, https://doi.org/10.2210/pdb9T1B/pdb, https://doi.org/10.2210/pdb9T1C/pdb, https://doi.org/10.2210/pdb9T1E/pdb, https://doi.org/10.2210/pdb9T1F/pdb, https://doi.org/10.2210/pdb9T1G/pdb, https://doi.org/10.2210/pdb9T1H/pdb, https://doi.org/10.2210/pdb9T1I/pdb, https://doi.org/10.2210/pdb9T1J/pdb, https://doi.org/10.2210/pdb9T0Y/pdb, https://doi.org/10.2210/pdb9T2J/pdb, https://doi.org/10.2210/pdb9T2K/pdb, https://doi.org/10.2210/pdb9T2L/pdb, https://doi.org/10.2210/pdb9T2M/pdb, https://doi.org/10.2210/pdb9T2N/pdb, https://doi.org/10.2210/pdb9T2O/pdb, https://doi.org/10.2210/pdb9T2P/pdb, https://doi.org/10.2210/pdb9T2Q/pdb, https://doi.org/10.2210/pdb9T2R/pdb, https://doi.org/10.2210/pdb9T2S/pdb, https://doi.org/10.2210/pdb9T6M/pdb, https://doi.org/10.2210/pdb9T8K/pdb, https://doi.org/10.2210/pdb9T8L/pdb, https://doi.org/10.2210/pdb9T8M/pdb, https://doi.org/10.2210/pdb9T8N/pdb, https://doi.org/10.2210/pdb9T8O/pdb, https://doi.org/10.2210/pdb9T8P/pdb, https://doi.org/10.2210/pdb9T8Q/pdb, https://doi.org/10.2210/pdb9T8R/pdb, https://doi.org/10.2210/pdb9T8S/pdb, https://doi.org/10.2210/pdb9T8T/pdb. The raw cryo-EM movies have been deposited in the EMPIAR database (https://www.ebi.ac.uk/empiar/) under the accession code EMPIAR-13296.
