# Supplementary material for: 23S rRNA modifications stimulate catalytic activity and prevent the formation of alternative structures
Source: Nucleic Acids Res. 2026 Aug 13;54(15):gkag800. doi: 10.1093/nar/gkag800 (PMC13469067; doi:10.1093/nar/gkag800)
Supplement: Supplementary file 1 [file gkag800_supplemental_file.pdf]

Supplementary material for

23S rRNA modifications stimulate catalytic activity and prevent the formation of alternative structures

Daniel S. D. Larsson[1,†], Aivar Liiv[2,†], Rya Ero[2], Jaanus Remme[2,*], Maria Selmer[1,*]

[1] Department of Cell and Molecular Biology, Uppsala University, BMC, P.O. Box 596, SE-75124 Uppsala, Sweden
[2] Institute of Molecular and Cell Biology, Riia 23, University of Tartu, 51010 Tartu, Estonia

* To whom correspondence should be addressed: Email: jaanus.remme@ut.ee and maria.selmer@icm.uu.se

† Daniel S.D. Larsson and Aivar Liiv contributed equally to this work.

Content:

Supplementary Tables 1-5

Supplementary Figures 1-26

Supplementary references

Supplementary Table 1. Modified nucleosides of 23S rRNA absent in the *E. coli* strains *Δ9* and *Δ10* and the corresponding modification enzymes**.**

23S rRNA position	Modification	Enzyme
955	Ψ	RluC
2069	m^7G	RlmKL (RlmK domain)
2251* (P-loop)	Gm	RlmB
2445	m^2G	RlmKL (RlmL domain)
2457*	Ψ	RluE
2498	Cm	RlmM
2501*	ho^5C	RlhA
2503	m^2A	RlmN
2504	Ψ	RluC
2552* (A-loop)	Um	RlmE**
2580	Ψ	RluC
2604	Ψ	RluF
2605	Ψ	RluB

*Evolutionarily conserved modification site from bacteria to humans

** Present in *Δ10* strain.

Supplementary Table 2. Temperature dependence of the rate (k_{obs}) of the Tripeptide assay (fMetPhe-tRNA + Pmn)

	Δ10		*Δ9*		WT	
Temp °C	k_{obs} sek^{-1}	std	k_{obs} sek^{-1}	std	k_{obs} sek^{-1}	std
15	1.83	0.38	3.08	0.32	5.23	0.39
20	2.28	0.55	3.85	0.30	7.85	1.00
25	5.45	0.93	5.50	0.45	9.91	1.27
30	6.50	0.73	6.90	0.41	16.01	2.18
37	7.49	0.93	8.42	1.03	25.89	3.02

Supplementary Table 3. Regions of interest in 23S rRNA

	Chain	Residues	Structure element#
PTCα	23S	2058–2063	H74
PTCβ	23S	2447–2453	H74
PTCɣ	23S	2499–2507	H89, H90
PTCδ	23S	2581–2587	H90
PTCε	23S	2607–2610	H93
P-loop	23S	2249–2255	H80
A-loop	23S	2552–2556	H92

Based on (1)

Supplementary Table 4. Class distribution after focused 3D classification of the tRNA sites.

	**APE**	**AP**	**PE**	**E**	**Total A-site**	**Total P-site**
**ΔRlmE**	52.6%	9.0%	24.0%	14.4%	61.6%	85.6%
**Δ9**	50.2%	–	34.9%	14.2%	50.2%	85.1%
**Δ10**	26.2%	–	49.6%	24.1%	26.2%	75.8%

Supplementary Table 5. Cryo-EM data collection, refinement and validation statistics

	Δ9 consensus (EMDB-55415) (PDB 9T0Y)	Δ9 class 0 (EMDB-55467) (PDB 9T2J)	Δ9 class 1 (EMDB-55468) (PDB 9T2K)	Δ9 class 2 (EMDB-55469) (PDB 9T2L)	Δ9 class 3 (EMDB-55470) (PDB 9T2M)	Δ9 class 4 (EMDB-55471) (PDB 9T2N)	Δ9 class 5 (EMDB-55472) (PDB 9T2O)	Δ9 class 6 (EMDB-55473) (PDB 9T2P)	Δ9 class 7 (EMDB-55474) (PDB 9T2Q)	Δ9 class 8 (EMDB-55475) (PDB 9T2R)	Δ9 class 9 (EMDB-55476) (PDB 9T2S)
**Data collection and processing**											
Magnification (kx)	165	165	165	165	165	165	165	165	165	165	165
Voltage (kV)	300	300	300	300	300	300	300	300	300	300	300
Electron exposure (e–/Å^2)	30.0	30.0	30.0	30.0	30.0	30.0	30.0	30.0	30.0	30.0	30.0
Defocus range (μm)	-0.6/-1.4	-0.6/-1.4	-0.6/-1.4	-0.6/-1.4	-0.6/-1.4	-0.6/-1.4	-0.6/-1.4	-0.6/-1.4	-0.6/-1.4	-0.6/-1.4	-0.6/-1.4
Pixel size (Å)	0.8215	0.8215	0.8215	0.8215	0.8215	0.8215	0.8215	0.8215	0.8215	0.8215	0.8215
Symmetry imposed	C1	C1	C1	C1	C1	C1	C1	C1	C1	C1	C1
Initial particle images (no.)	1,046,157	1,046,157	1,046,157	1,046,157	1,046,157	1,046,157	1,046,157	1,046,157	1,046,157	1,046,157	1,046,157
Final particle images (no.)	910,722	107,941	100,949	93,493	88,813	88,625	88,358	87,602	87,185	84,723	83,033
Map resolution (Å)	1.87	2.02	2.06	2.07	2.06	2.06	2.07	2.08	2.08	2.22	2.07
FSC threshold	0.143	0.143	0.143	0.143	0.143	0.143	0.143	0.143	0.143	0.143	0.143
Map sharpening *B* factor (Å^2)	-30.50	-35.90	-38.50	-38.20	-37.10	-37.50	-36.80	-37.80	-38.40	-39.50	-37.40
**Refinement**											
Initial model(s) used	9SYH	9T0Y	9T0Y	9T0Y	9T0Y	9T0Y	9T0Y	9T0Y	9T0Y	9T0Y	9T0Y
Model resolution (Å)	1.85	2.05	2.07	2.08	2.08	2.08	2.08	2.09	2.09	2.24	2.09
FSC threshold	0.5	0.5	0.5	0.5	0.5	0.5	0.5	0.5	0.5	0.5	0.5
Model composition											
Non-hydrogen atoms	151,117	14,602	15,318	15,318	15,018	15,196	14,939	15,164	15,318	15,019	15,316
Protein residues	5,553	204	212	212	206	197	196	195	212	206	212
RNA residues	4,731	600	631	631	619	631	619	630	631	619	631
B factors (Å^2)	83.37	49.73	52.06	53.29	53.05	56.15	55.46	56.73	53.55	33.44	57.02
Protein	84.21	57.82	56.50	52.92	56.34	52.20	52.72	51.79	54.88	38.07	60.41
RNA	84.79	48.42	51.30	53.14	52.41	56.39	55.54	57.08	53.17	32.62	56.41
R.m.s. deviations											
Bond lengths (Å)	0.0101	0.0087	0.0089	0.0088	0.0088	0.0088	0.0088	0.0088	0.0088	0.0089	0.0089
Bond angles (°)	1.75	1.72	1.71	1.71	1.72	1.71	1.72	1.71	1.70	1.72	1.71
Validation											
MolProbity score	0.93	1.31	1.21	1.29	1.33	1.35	1.33	1.27	1.27	1.31	1.28
Clashscore	0.90	1.84	1.59	1.84	2.31	2.62	2.11	1.99	1.72	2.14	1.76
Poor rotamers (%)	0.07	0.65	0.00	0.00	0.00	0.00	0.68	0.00	0.00	0.00	0.00
Ramachandran plot											
Favored (%)	97.11	94.77	95.56	95.00	95.40	95.71	95.06	95.65	95.00	95.40	95.00
Disallowed (%)	0.09	0.00	0.00	0.00	0.00	0.00	0.00	0.00	0.00	0.00	0.00

Supplementary Table 5 Cryo-EM data collection, refinement and validation statistics (Continued)

	Δ10 consensus (EMDB-55618) (PDB 9T6M)	Δ10 class 0 (EMDB-55702) (PDB 9T8K)	Δ10 class 1 (EMDB-55703) (PDB 9T8L)	Δ10 class 2 (EMDB-55704) (PDB 9T8M)	Δ10 class 3 (EMDB-55705) (PDB 9T8N)	Δ10 class 4 (EMDB-55706) (PDB 9T8O)	Δ10 class 5 (EMDB-55707) (PDB 9T8P)	Δ10 class 6 (EMDB-55708) (PDB 9T8Q)	Δ10 class 7 (EMDB-55709) (PDB 9T8R)	Δ10 class 8 (EMDB-55710) (PDB 9T8S)	Δ10 class 9 (EMDB-55711) (PDB 9T8T)
**Data collection and processing**											
Magnification (kx)	165	165	165	165	165	165	165	165	165	165	165
Voltage (kV)	300	300	300	300	300	300	300	300	300	300	300
Electron exposure (e–/Å²)	30.0	30.0	30.0	30.0	30.0	30.0	30.0	30.0	30.0	30.0	30.0
Defocus range (μm)	-0.6/-1.4	-0.6/-1.4	-0.6/-1.4	-0.6/-1.4	-0.6/-1.4	-0.6/-1.4	-0.6/-1.4	-0.6/-1.4	-0.6/-1.4	-0.6/-1.4	-0.6/-1.4
Pixel size (Å)	0.8215	0.8215	0.8215	0.8215	0.8215	0.8215	0.8215	0.8215	0.8215	0.8215	0.8215
Symmetry imposed	C1	C1	C1	C1	C1	C1	C1	C1	C1	C1	C1
Initial particle images (no.)	863,376	863,376	863,376	863,376	863,376	863,376	863,376	863,376	863,376	863,376	863,376
Final particle images (no.)	582,240	61,890	59,988	59,781	58,711	58,616	57,630	57,497	56,658	56,208	55,261
Map resolution (Å)	1.87	2.18	2.21	2.20	2.22	2.19	2.22	2.23	2.22	2.22	2.23
FSC threshold	0.143	0.143	0.143	0.143	0.143	0.143	0.143	0.143	0.143	0.143	0.143
Map sharpening *B* factor (Å²)	-25.24	-38.30	-39.20	-38.90	-39.90	-38.50	-39.60	-39.90	-39.40	-39.80	-39.90
**Refinement**											
Initial model(s) used	9T0Y	9T6M	9T6M	9T6M	9T6M	9T6M	9T6M	9T6M	9T6M	9T6M	9T6M
Model resolution (Å)	1.85	2.22	2.25	2.23	2.25	2.25	2.26	2.26	2.25	2.25	2.26
FSC threshold	0.5	0.5	0.5	0.5	0.5	0.5	0.5	0.5	0.5	0.5	0.5
Model composition											
Non-hydrogen atoms	150,408	14,213	14,339	14,222	15,049	14,255	14,684	14,741	14,575	14,706	14,937
Protein residues	5,553	184	193	187	203	196	196	195	193	197	203
RNA residues	4,724	594	596	593	626	591	611	614	607	612	621
B factors (Å²)	87.77	52.86	26.19	25.68	61.18	52.79	58.01	30.94	29.65	58.42	60.59
Protein	91.40	55.66	33.14	29.28	62.60	61.26	60.23	34.32	33.10	61.92	62.93
RNA	88.26	52.38	25.21	25.09	60.91	51.59	57.63	30.42	29.12	57.88	60.23
R.m.s. deviations											
Bond lengths (Å)	0.0102	0.0089	0.0089	0.0089	0.0089	0.0089	0.0089	0.0090	0.0089	0.0089	0.0090
Bond angles (°)	1.78	1.73	1.73	1.73	1.74	1.74	1.72	1.73	1.73	1.73	1.74
Validation											
MolProbity score	0.94	1.14	1.38	1.23	1.32	1.21	1.28	1.30	1.25	1.35	1.27
Clashscore	0.96	1.40	1.79	1.62	2.26	1.39	2.14	1.70	1.63	1.87	1.89
Poor rotamers (%)	0.07	0.00	1.37	0.00	0.66	0.00	0.67	0.00	0.00	0.67	0.66
Ramachandran plot											
Favored (%)	97.11	96.05	95.09	95.48	95.38	95.18	95.78	94.55	95.09	94.01	95.38
Disallowed (%)	0.09	0.00	0.00	0.00	0.00	0.00	0.00	0.00	0.00	0.00	0.00

Supplementary Table 5 Cryo-EM data collection, refinement and validation statistics (Continued)

	ΔRlmE consensus (EMDB-55349) (PDB 9SYH)	ΔRlmE class 0 (EMDB-55427) (PDB 9T19)	ΔRlmE class 1 (EMDB-55428) (PDB 9T1A)	ΔRlmE class 2 (EMDB-55429) (PDB 9T1B)	ΔRlmE class 3 (EMDB-55430) (PDB 9T1C)	ΔRlmE class 4 (EMDB-55432) (PDB 9T1E)	ΔRlmE class 5 (EMDB-55433) (PDB 9T1F)	ΔRlmE class 6 (EMDB-55434) (PDB 9T1G)	ΔRlmE class 7 (EMDB-55435) (PDB 9T1H)	ΔRlmE class 8 (EMDB-55436) (PDB 9T1I)	ΔRlmE class 9 (EMDB-55437) (PDB 9T1J)
**Data collection and processing**											
Magnification (kx)	105	105	105	105	105	105	105	105	105	105	105
Voltage (kV)	300	300	300	300	300	300	300	300	300	300	300
Electron exposure (e–/Å^2)	40.6	40.6	40.6	40.6	40.6	40.6	40.6	40.6	40.6	40.6	40.6
Defocus range (µm)	-0.4/-1.0	-0.4/-1.0	-0.4/-1.0	-0.4/-1.0	-0.4/-1.0	-0.4/-1.0	-0.4/-1.0	-0.4/-1.0	-0.4/-1.0	-0.4/-1.0	-0.4/-1.0
Pixel size (Å)	0.8228	0.8228	0.8228	0.8228	0.8228	0.8228	0.8228	0.8228	0.8228	0.8228	0.8228
Symmetry imposed	C1	C1	C1	C1	C1	C1	C1	C1	C1	C1	C1
Initial particle images (no.)	736,925	736,925	736,925	736,925	736,925	736,925	736,925	736,925	736,925	736,925	736,925
Final particle images (no.)	513,497	46,784	54,635	53,040	15,832	57,081	55,766	56,652	51,232	53,129	69,346
Map resolution (Å)	1.89	2.41	2.39	2.32	3.07	2.28	2.32	2.29	2.33	2.30	2.23
FSC threshold	0.143	0.143	0.143	0.143	0.143	0.143	0.143	0.143	0.143	0.143	0.143
Map sharpening *B* factor (Å^2)	-44.2	-39.60	-39.70	-40.30	-15.40	-38.80	-41.00	-40.00	-39.60	-40.00	-38.70
**Refinement**											
Initial model(s) used	8CF1, 8CGJ, 8CGK, 7K00	9SYH	9SYH	9SYH	9SYH	9SYH	9SYH	9SYH	9SYH	9SYH	9SYH
Model resolution (Å)	2.07	2.55	2.41	2.45	3.27	2.40	2.40	2.40	2.41	2.40	2.32
FSC threshold	0.5	0.5	0.5	0.5	0.5	0.5	0.5	0.5	0.5	0.5	0.5
Model composition											
Non-hydrogen atoms	151,069	15,294	15,298	15,254	14,819	15,297	15,295	15,038	15,256	15,041	15,298
Protein residues	5,553	211	211	205	203	211	211	211	205	211	211
RNA residues	4,734	629	629	629	617	629	629	617	629	617	629
B factors (Å^2)	104.74	31.14	25.23	27.35	88.81	29.08	26.47	24.97	29.43	25.73	28.35
Protein	110.26	31.59	25.82	29.15	87.97	27.81	26.73	28.51	27.11	28.51	27.06
RNA	104.56	30.90	24.92	26.88	88.95	29.05	26.23	24.29	29.50	25.13	28.32
R.m.s. deviations											
Bond lengths (Å)	0.0094	0.0094	0.0095	0.0093	0.0098	0.0094	0.0094	0.0093	0.0094	0.0094	0.0094
Bond angles (°)	1.69	1.81	1.79	1.78	1.85	1.77	1.79	1.79	1.79	1.79	1.80
Validation											
MolProbity score	0.90	1.33	1.43	1.39	1.44	1.24	1.28	1.34	1.41	1.39	1.32
Clashscore	0.82	2.73	2.22	2.48	2.59	2.01	2.01	1.92	2.19	2.30	2.01
Poor rotamers (%)	0.13	0.64	1.27	0.65	0.65	0.00	0.00	0.64	0.65	0.64	0.64
Ramachandran plot											
Favored (%)	97.20	96.09	94.97	94.74	94.15	96.09	95.53	94.41	93.64	94.41	94.97
Disallowed (%)	0.09	0.00	0.00	0.00	0.00	0.00	0.00	0.00	0.00	0.00	0.00

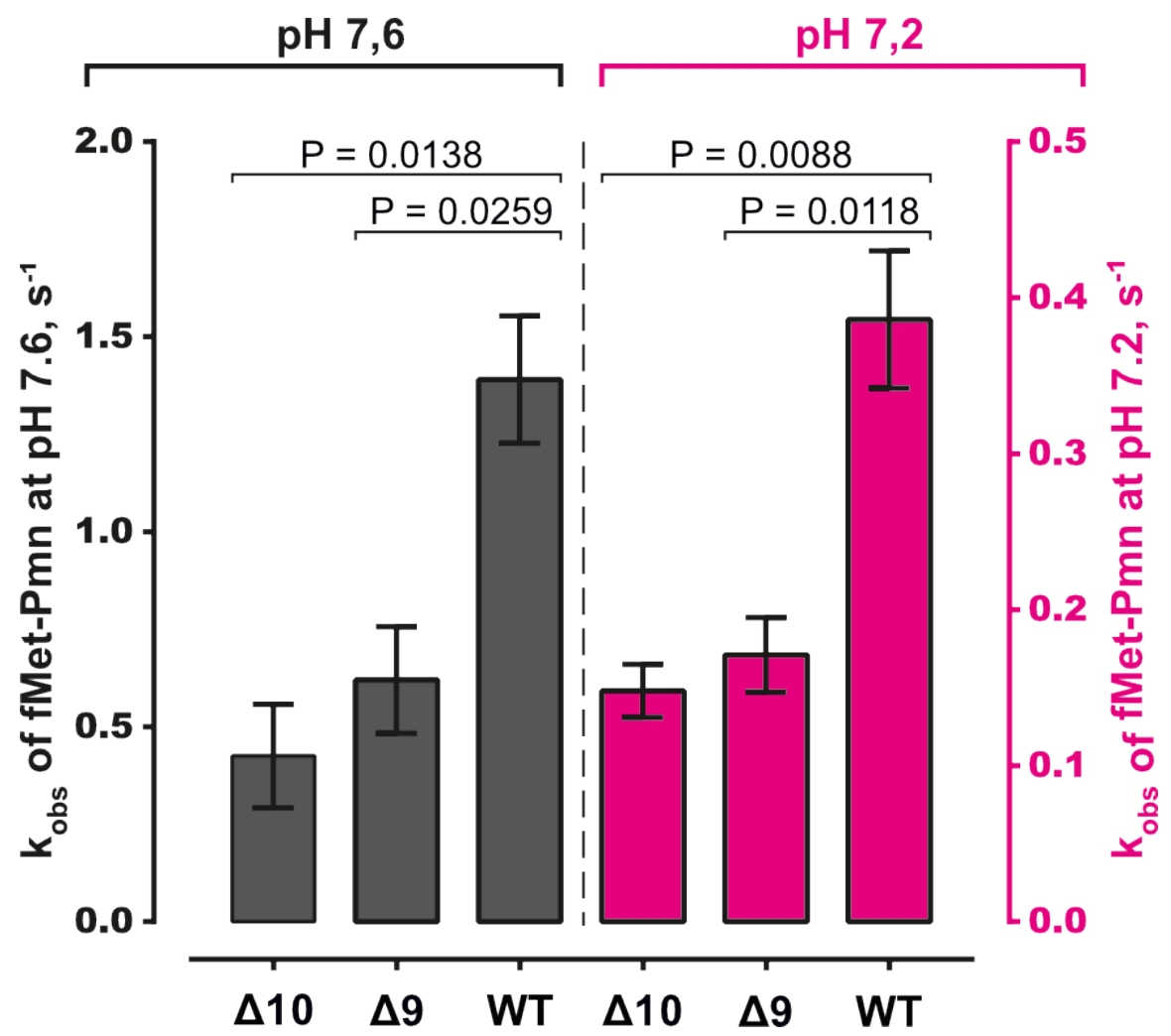

Supplementary Figure 1. The effect of pH on the puromycin reaction with fMet-tRNA (dipeptide assay) is similar for WT and hypo-modified ribosomes.

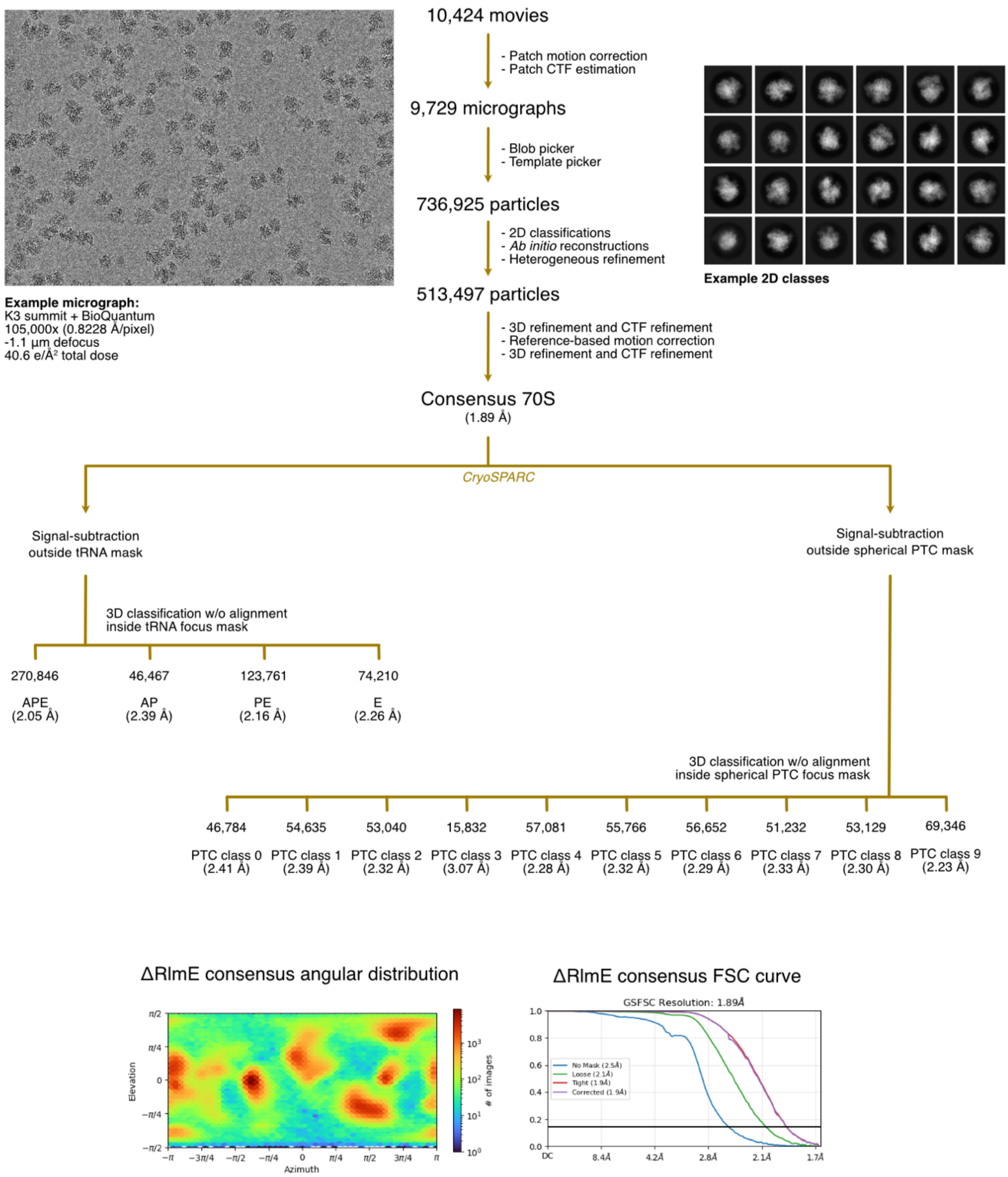

Supplementary Figure 2. Cryo-EM single-particle analysis workflow for the ΔRlmE reconstructions. Example micrograph, example 2D classes, as well as Euler angle distribution and FSC curve for consensus reconstruction.

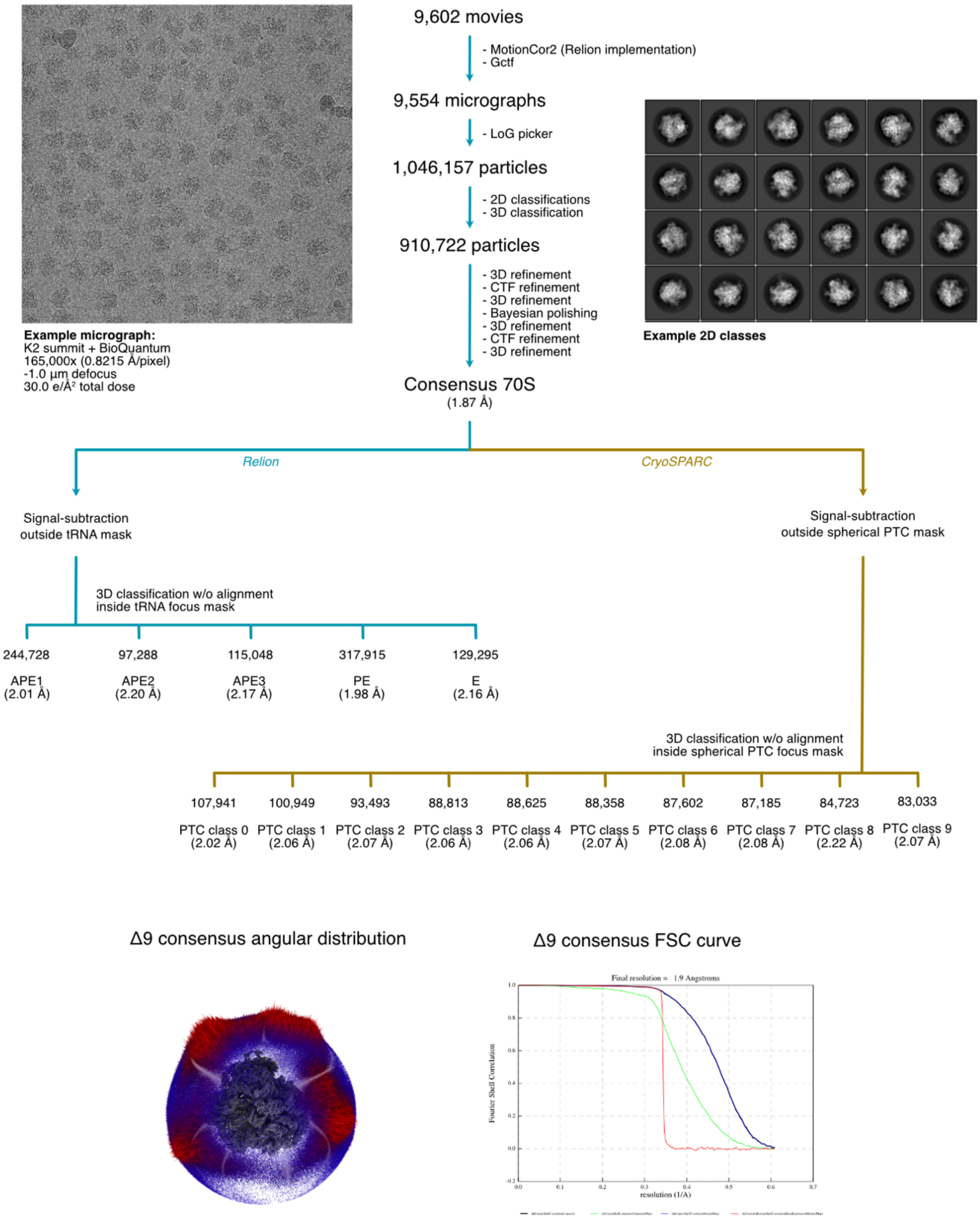

Supplementary Figure 3. Cryo-EM single-particle analysis workflow for the Δ9 reconstructions. Example micrograph, example 2D classes, as well as Euler angle distribution and FSC curve for consensus reconstruction.

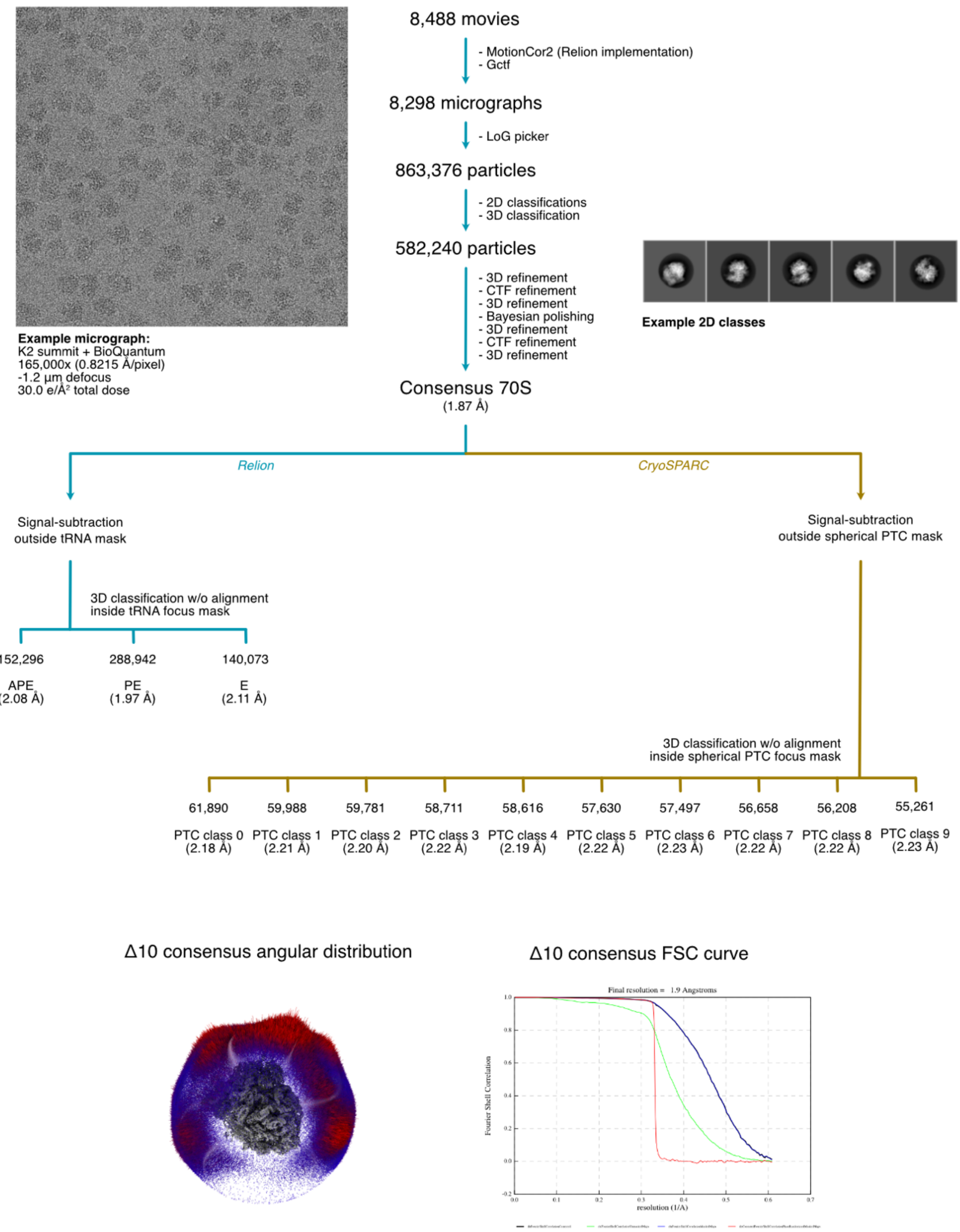

Supplementary Figure 4. Cryo-EM single-particle analysis workflow for the Δ10 reconstructions. Example micrograph, example 2D classes, as well as Euler angle distribution and FSC curve for consensus reconstruction.

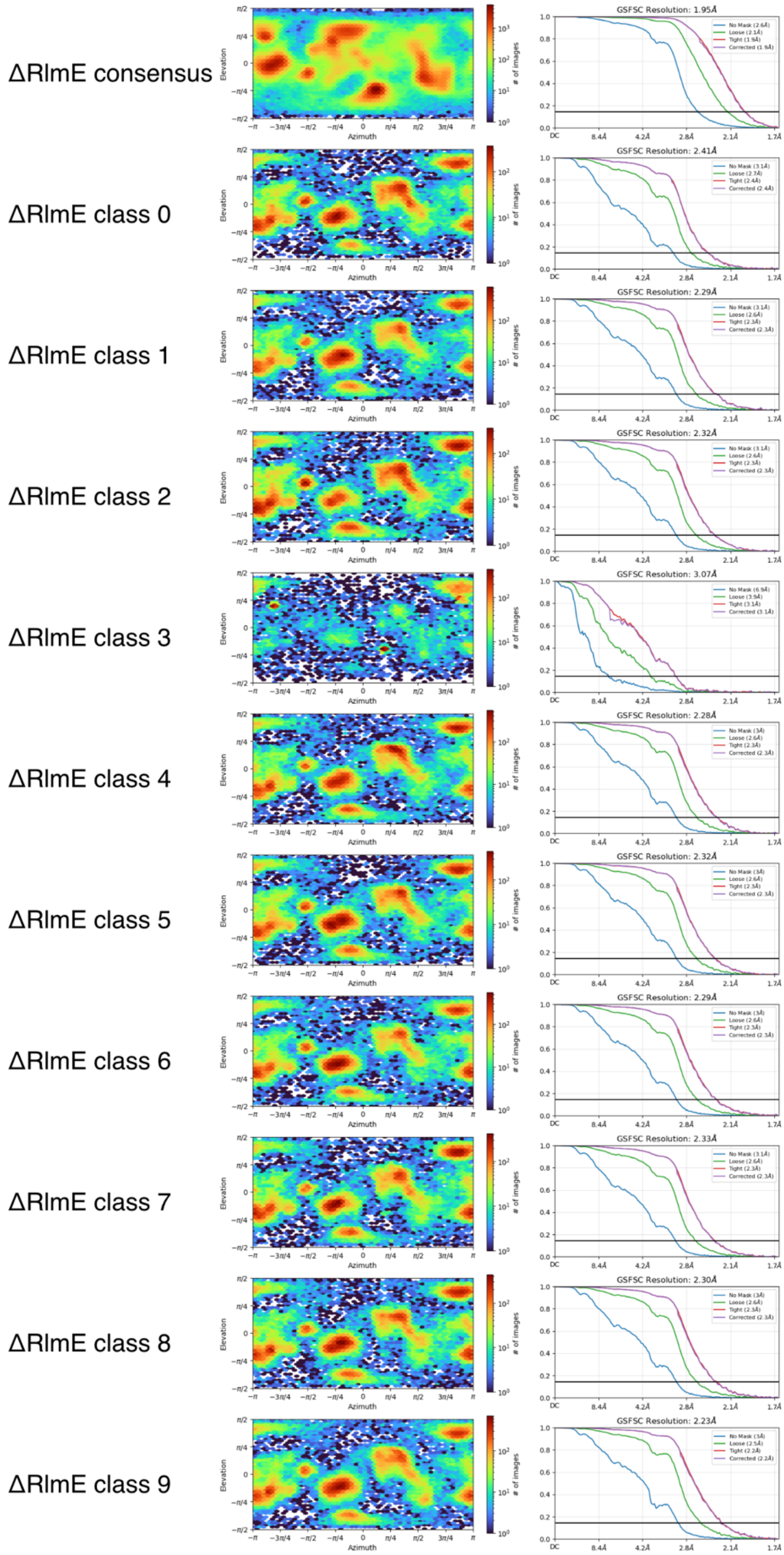

Supplementary Figure 5. Euler angle distribution and FSC curve for each ΔRlmE class.

Δ9 class 0

Δ9 class 1

Δ9 class 2

Δ9 class 3

Δ9 class 4

Δ9 class 5

Δ9 class 6

Δ9 class 7

Δ9 class 8

Δ9 class 9

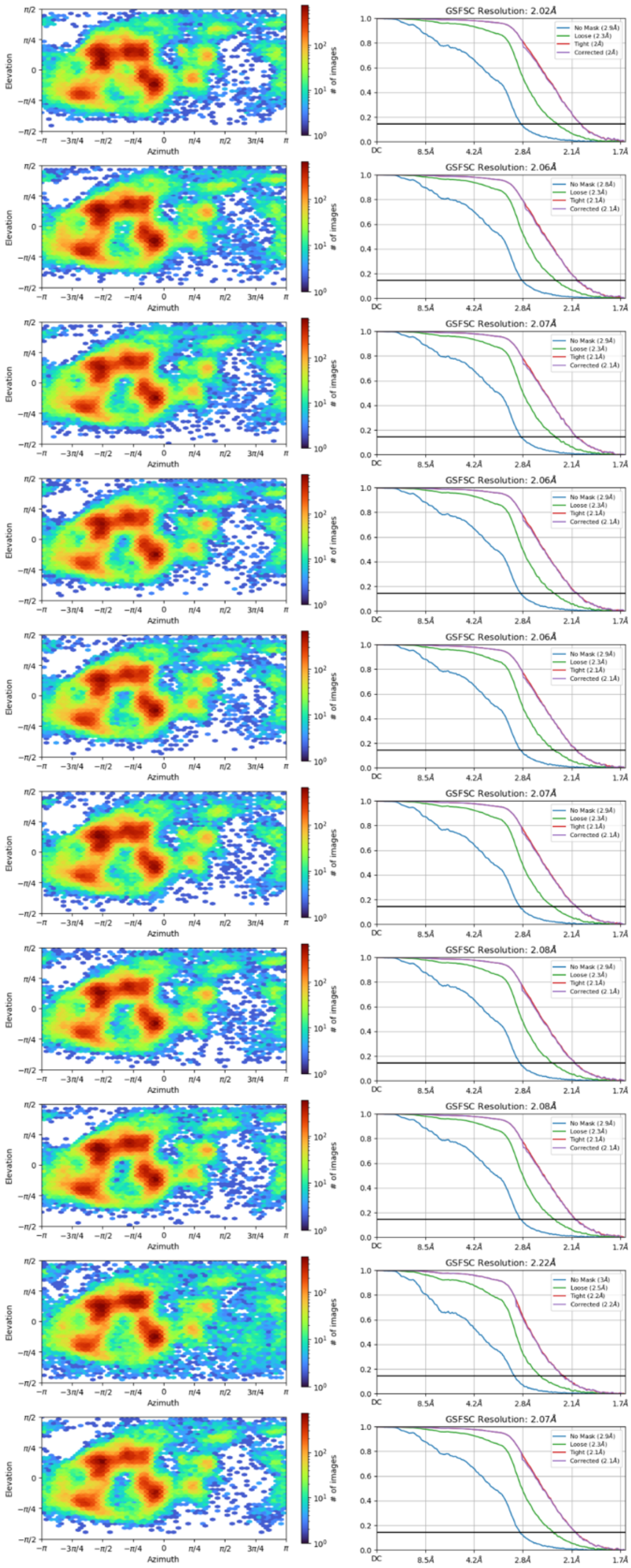

Supplementary Figure 6. Euler angle distribution and FSC curve for each Δ9 class.

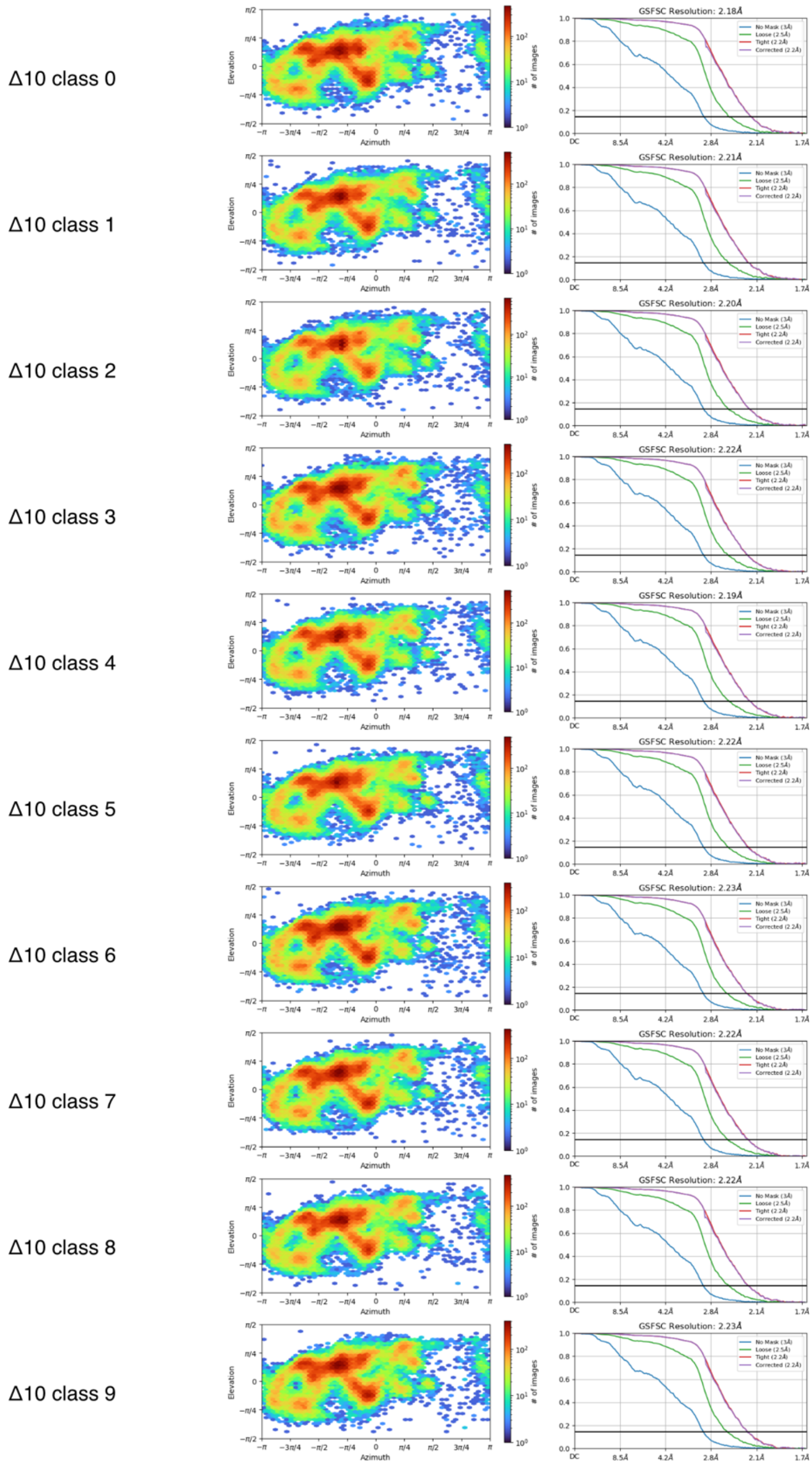

Supplementary Figure 7. Euler angle distribution and FSC curve for each Δ10 class.

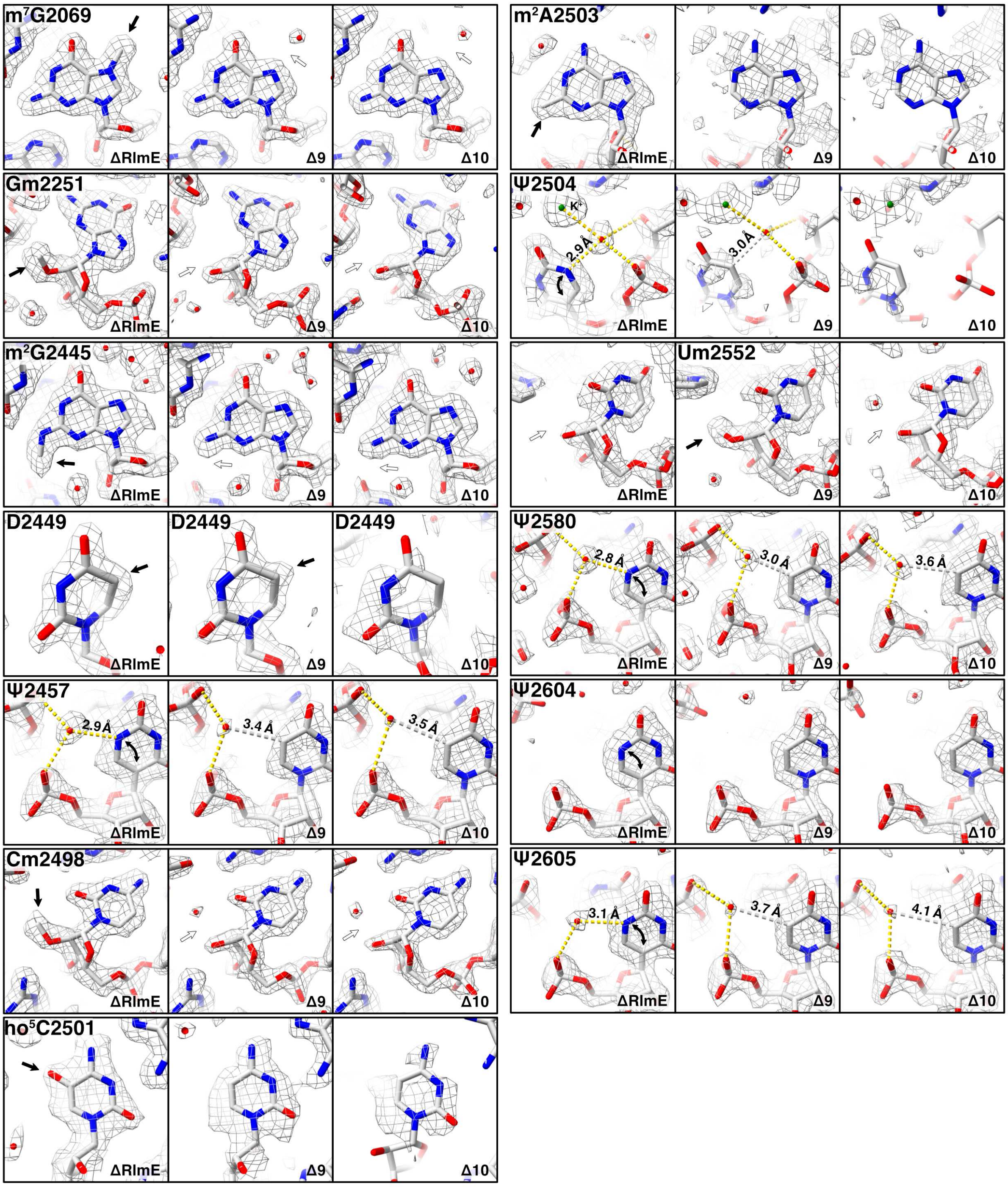

Supplementary Figure 8. Map support for post-transcriptional modifications in consensus reconstructions. Black arrows indicate the presence and white arrows the absence of modifications.

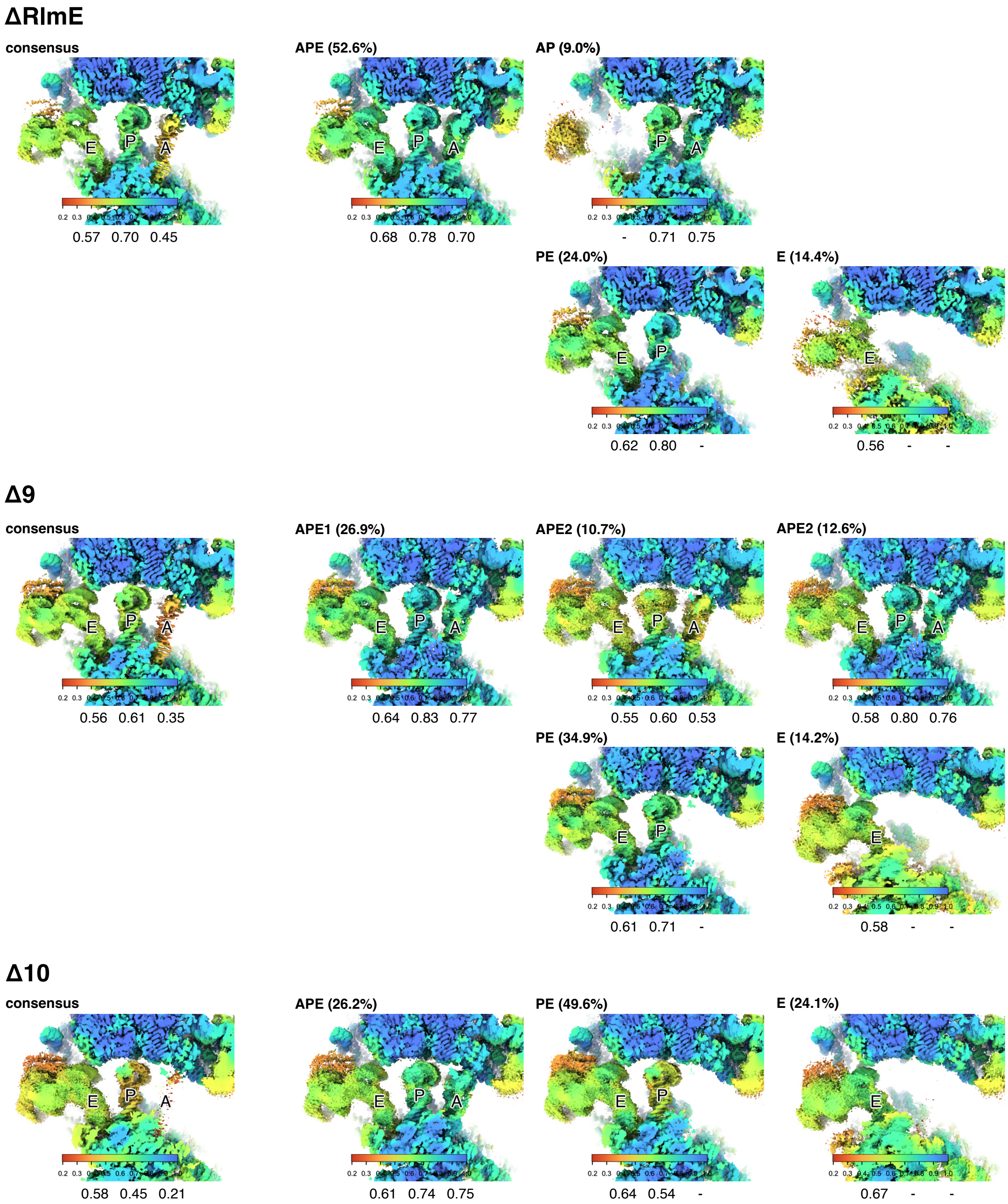

Supplementary Figure 9. tRNA occupancy analysis for consensus reconstructions and tRNA classes using Occupy (3). Colours represent the estimated local occupancy. Values below each figure represent the occupancy at the middle of each tRNA molecule. Percentages indicate the fraction of particles in that class.

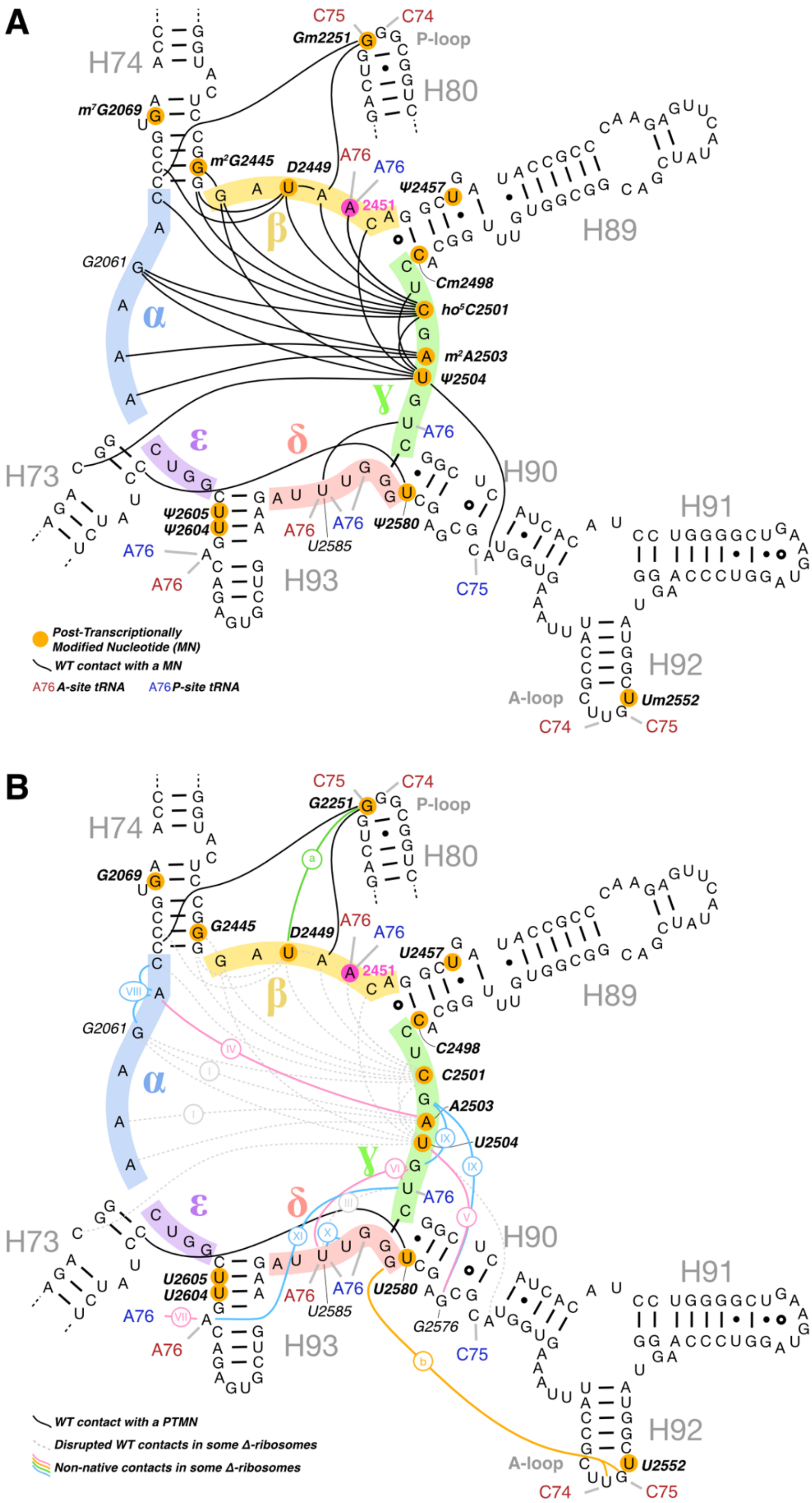

Supplementary Figure 10. Tertiary contacts in the PTC region. **A**. Secondary structure diagram as in Figure 1B with tertiary contacts from post-transcriptionally modified nucleotides in the WT ribosome (PDB 8EMM) indicated with black lines. **B**. Disrupted native contacts (dashed grey lines) and non-native contacts (colours) observed in some Δ9 and Δ10 classes. Roman numbers I–XI as shown in Figure 7. Contact a (green) represents the non-native hydrogen bond in Figure 5L. Contact b (orange) represents the stack in Figure 6D.

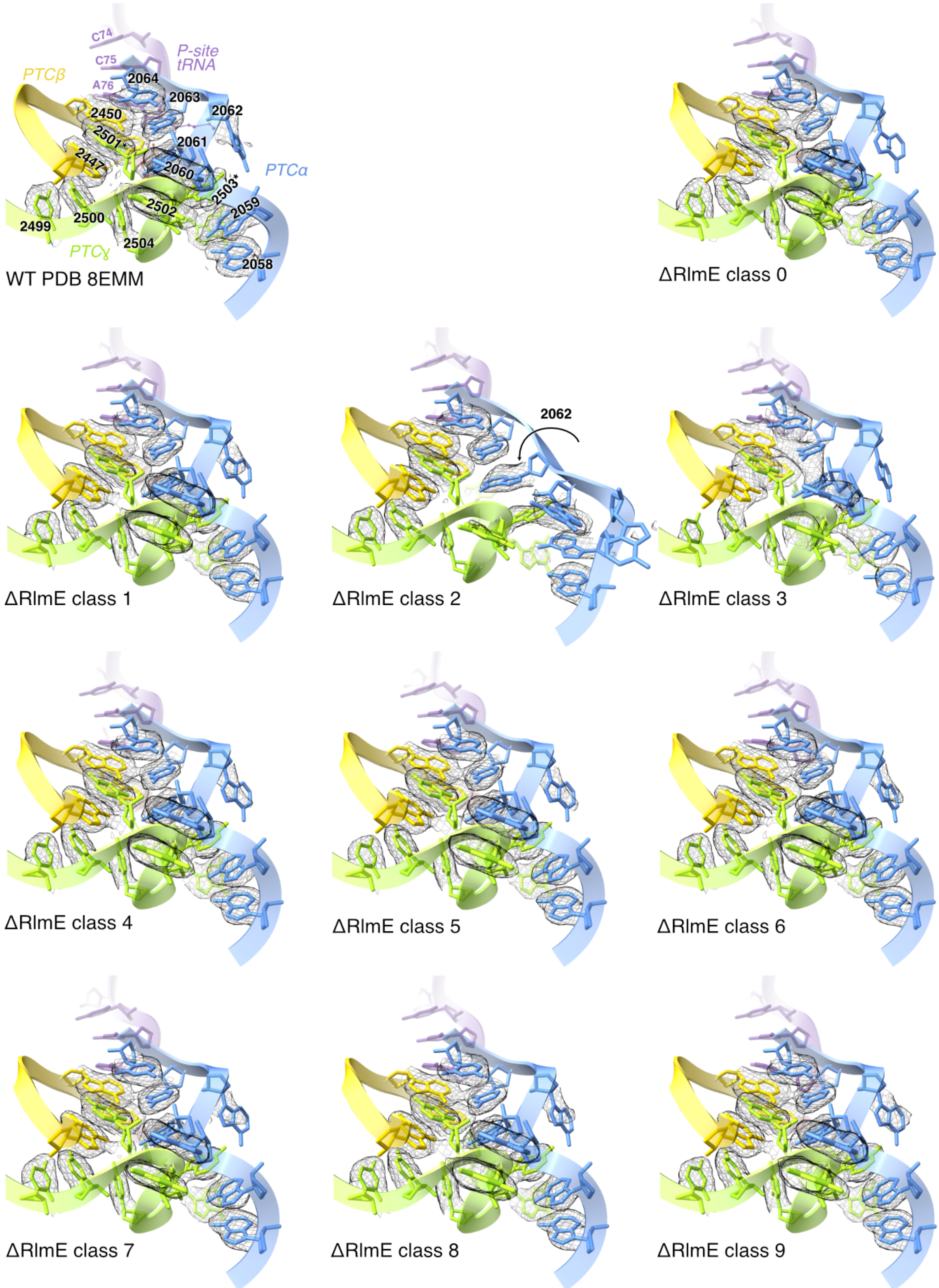

Supplementary Figure 11. Conformations of PTCα (blue) and PTCγ (green) in WT (PDB ID 8EMM) and ΔRlmE classes. Segmented maps are shown as mesh within 2 Å of bases.

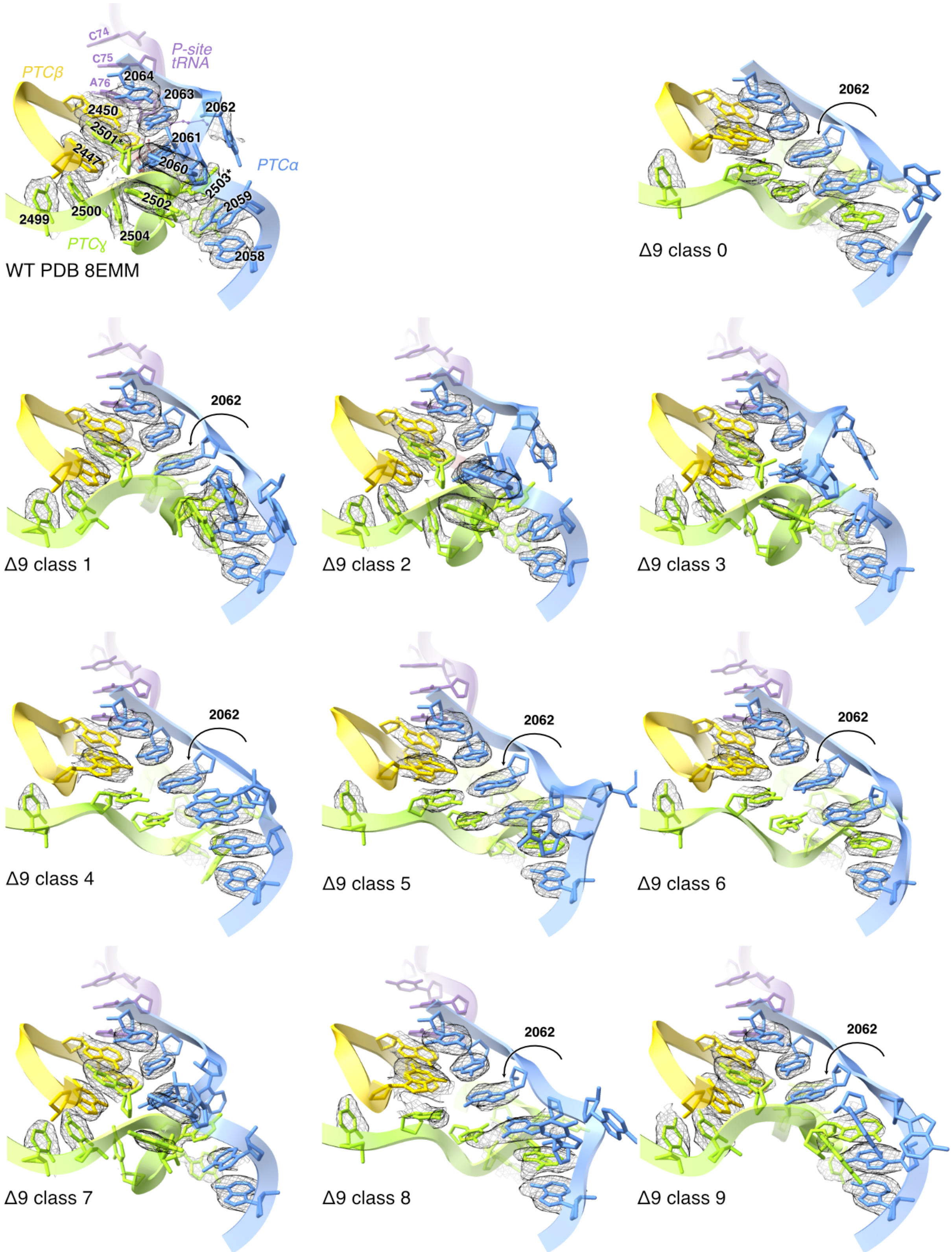

Supplementary Figure 12. Conformations of PTCα (blue) and PTCγ (green) in WT (PDB ID 8EMM) and Δ9 classes. Segmented maps are shown as mesh within 2 Å of bases.

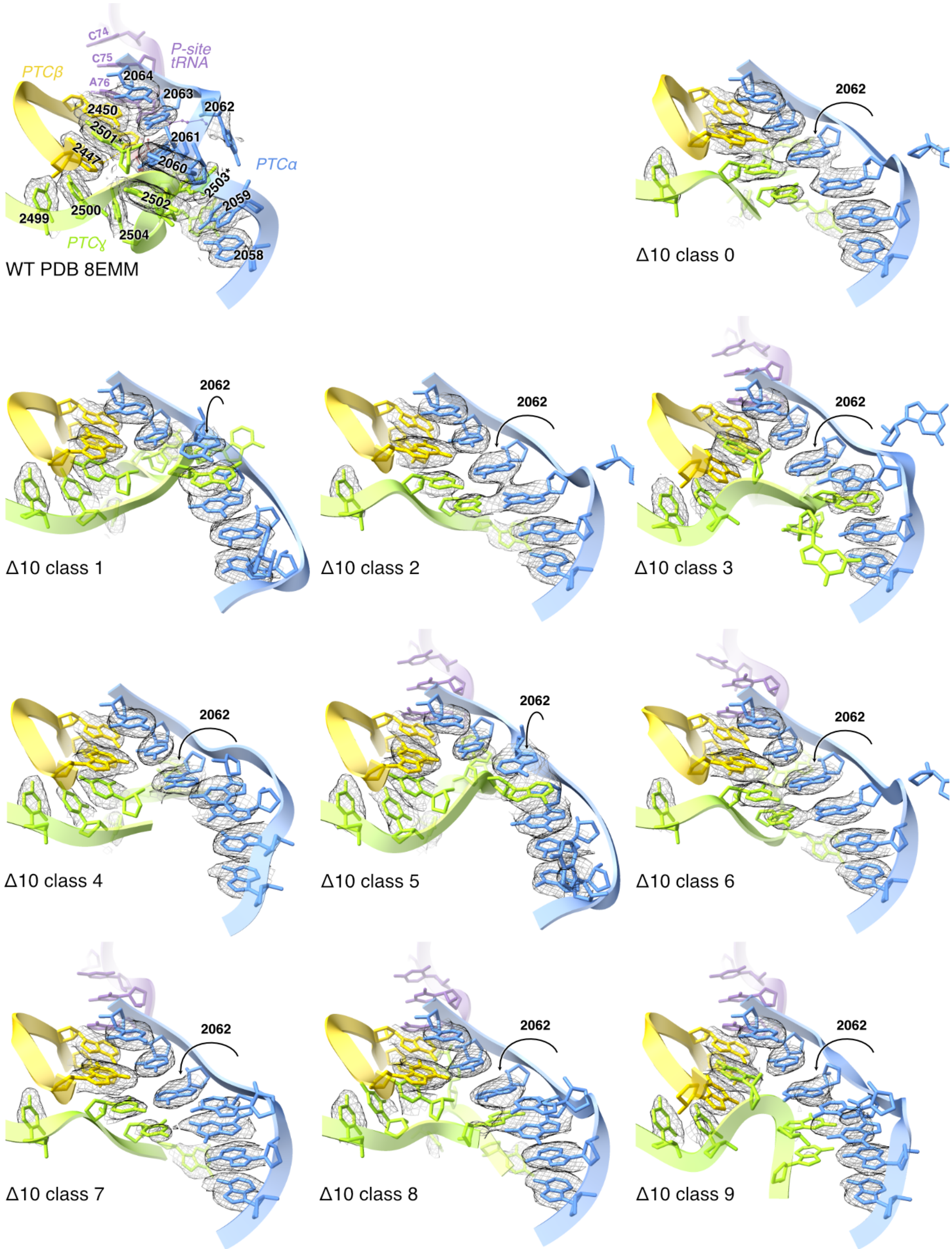

Supplementary Figure 13. Conformations of PTCα (blue) and PTCγ (green) in WT (PDB ID 8EMM) and Δ10 classes. Segmented maps are shown as mesh within 2 Å of bases.

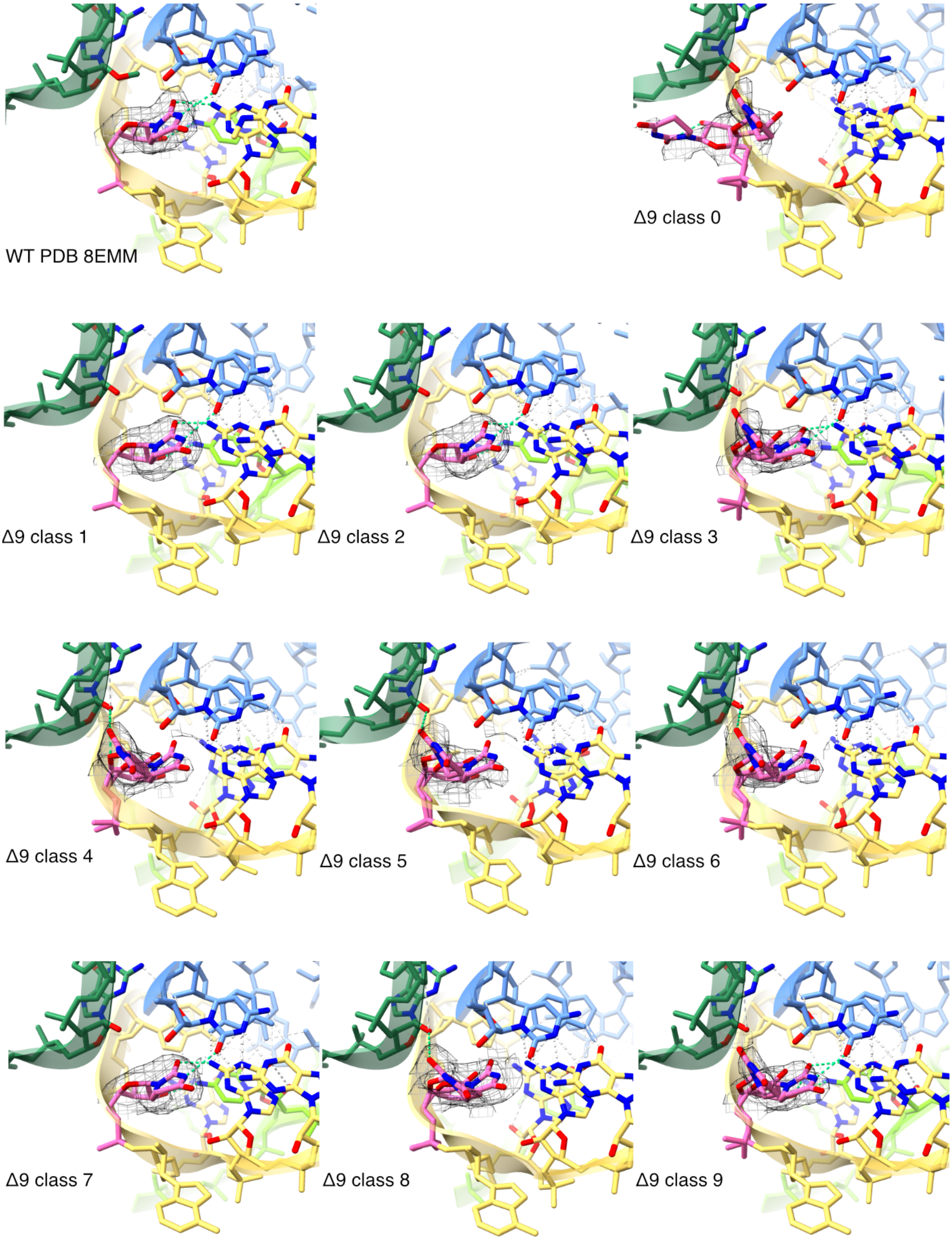

Supplementary Figure 14. Conformation of D2449 (pink) in Δ9 classes with PTCα (blue), PTCβ (yellow), C2501 (lime green) and G2251 (dark green). The D2449 base is rotated in classes 0, 3, 4, 5, 6, 8 and 9 (Figure 6L). In Δ9 class 0, D2449 shows an additional alternative conformation. Segmented map is shown as mesh within 2 Å of D2449.

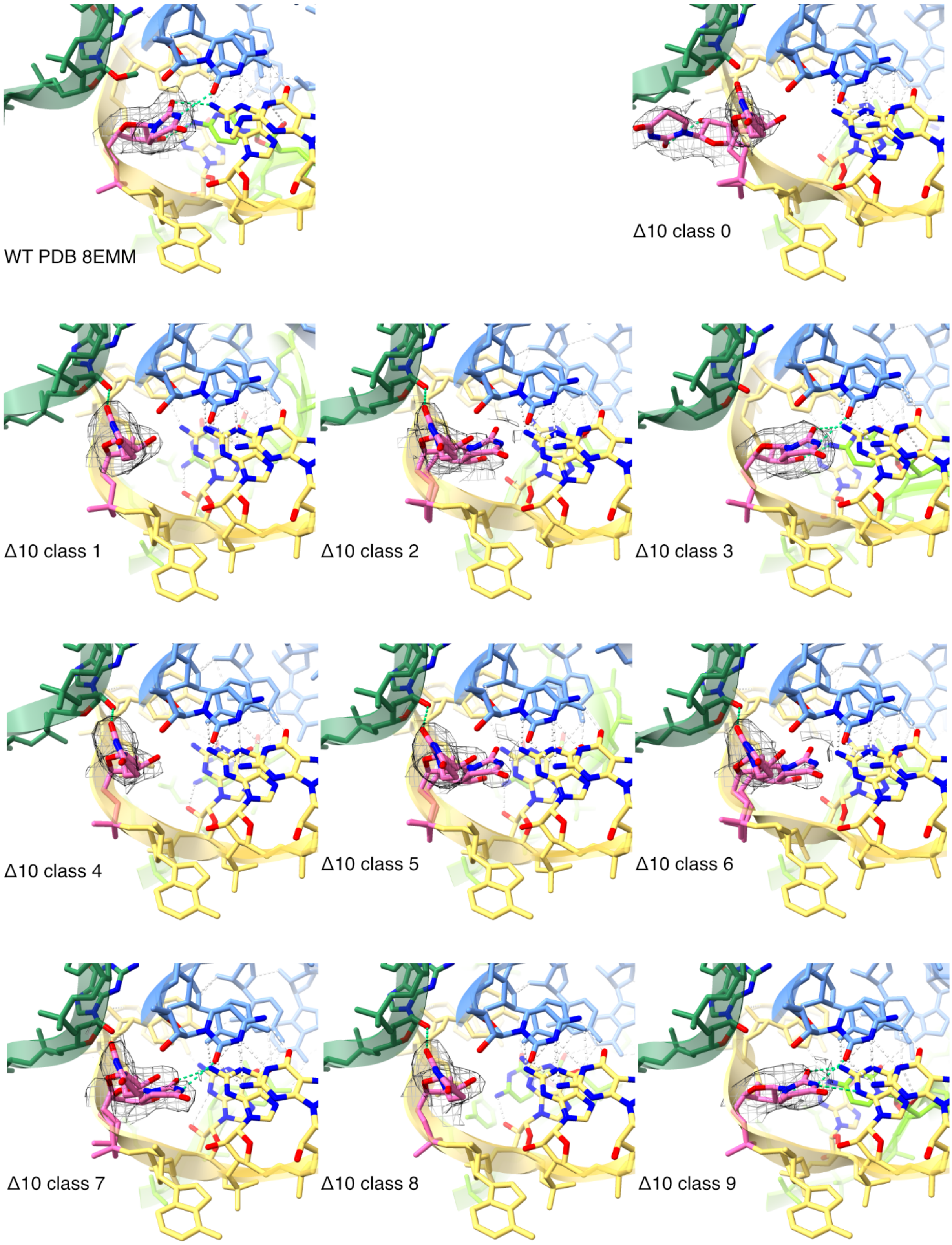

Supplementary Figure 15. Conformation of D2449 (pink) in Δ10 classes with PTCα (blue), PTCβ (yellow), C2501 (lime green) and G2251 (dark green). The base is rotated in classes 0, 1, 2, 4, 5, 6, 7, 8 (Figure 6L). In Δ10 class 0, D2449 shows an additional alternative conformation. Segmented map is shown as mesh within 2 Å of D2449.

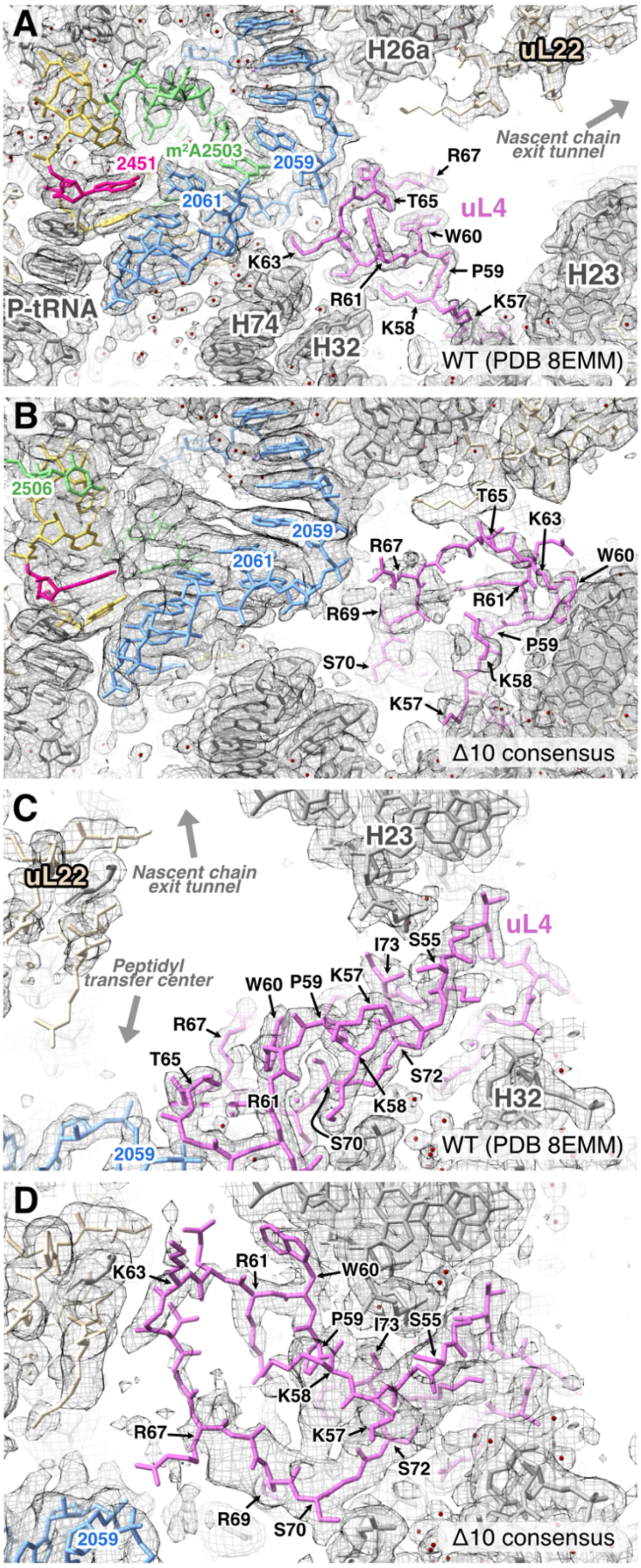

Supplementary Figure 16. Misfolded PTCα and alternative folding of the 55–73 loop in r-protein uL4. **A.** Wild-type fold of uL4 (purple) in contact with PTCα (blue) at nucleotide A2059. The catalytic residue A2451 is shown in pink. The P-site tRNA is located to the left and the nascent chain exit tunnel extends to the top right. **B.** Misfolded uL4 conformation, partially obstructing the exit tunnel in the consensus Δ10 structure. Same view as in A. **C.** Wild-type conformation shown from another angle with the PTC toward the lower left and the exit of the tunnel towards the top. **D.** Δ10 structure in the same view as in C.

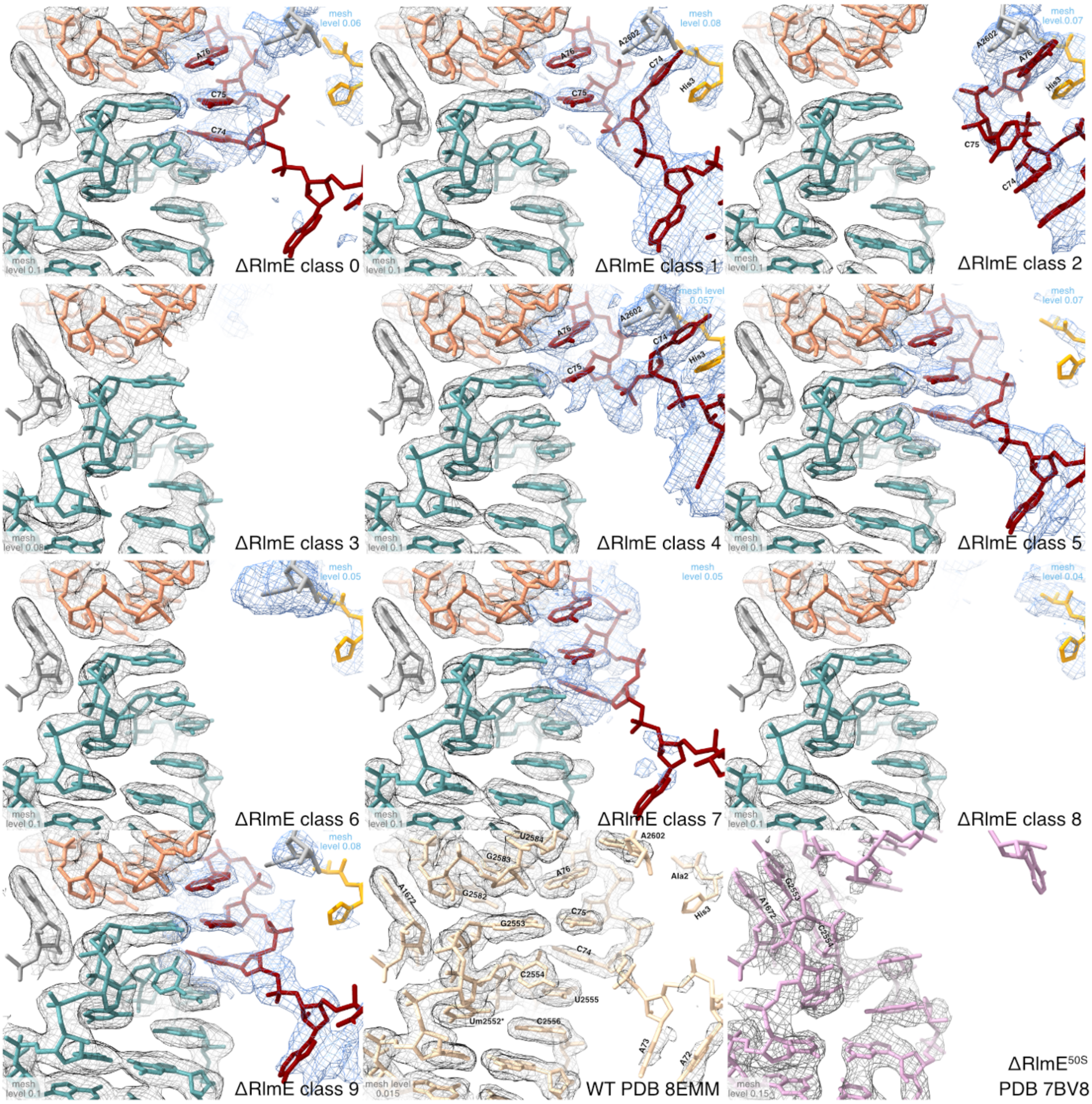

Supplementary Figure 17. A-loop conformation for ΔRlmE classes. The view and colouring the same as in Figure 6. Reference structures are WT 70S with A- and P-site tRNA (PDB 8EMM (2)) and 50S ΔRlmE (PDB 7BV8 (4)).

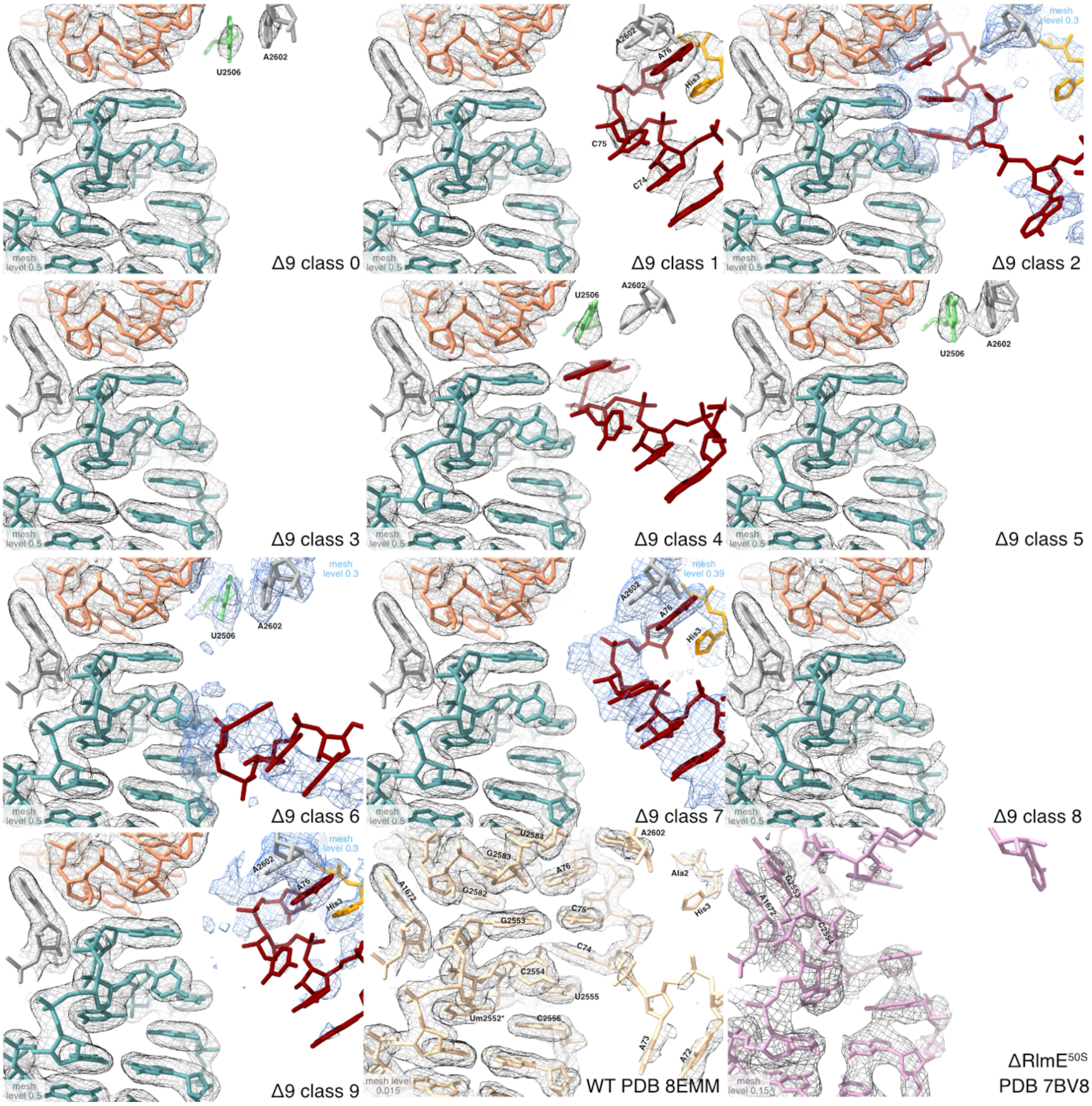

Supplementary Figure 18. A-loop conformation for Δ9 classes. The view and colouring the same as in Figure 6. Reference structures are WT 70S with A- and P-site tRNA (PDB 8EMM (2)) and 50S ΔRlmE (PDB 7BV8 (4)).

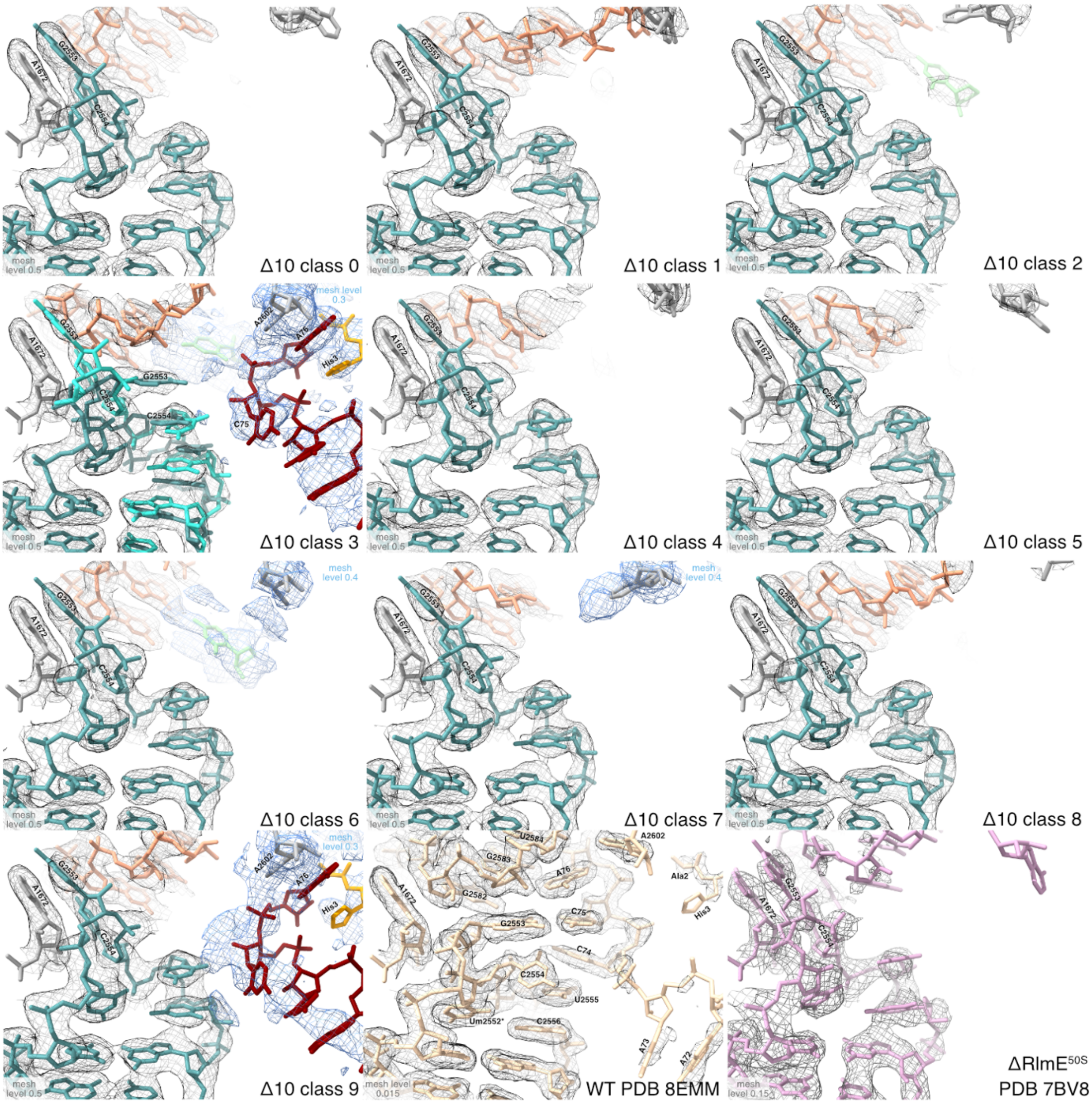

Supplementary Figure 19. A-loop conformation for Δ10 classes. The view and colouring the same as in Figure 6. Class 3 has two alternate conformations for the A-loop, one wild-type like (green) and one misfolded (cyan). Reference structures are WT 70S with A- and P-site tRNA (PDB 8EMM (2)) and 50S ΔRlmE (PDB 7BV8 (4)).

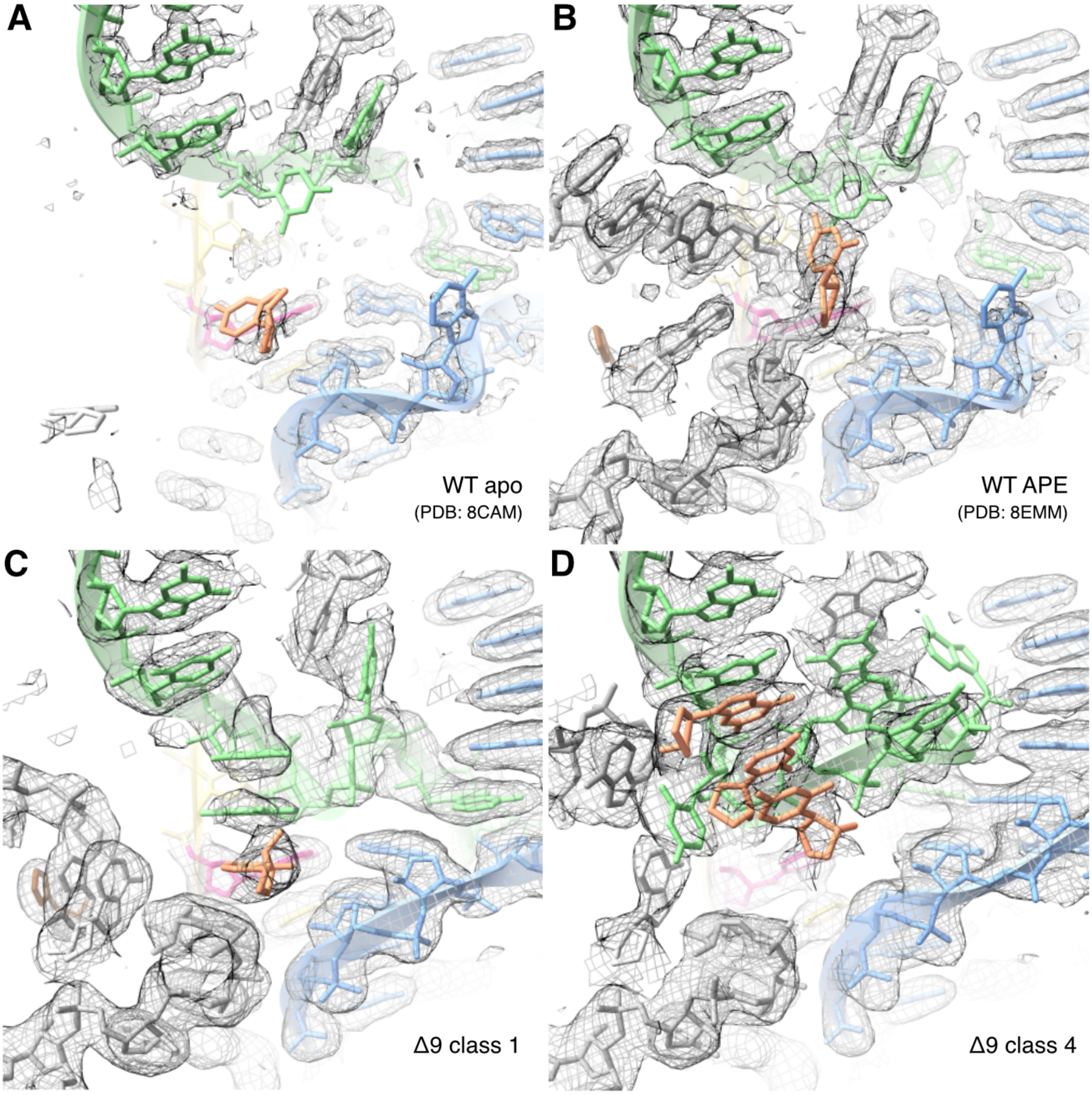

Supplementary Figure 20. Alternative stacking at the PTC. Same views as in Figure 7. Segmented maps are shown as mesh within 2 Å of residues in any of the classes. **A.** WT conformation with no bound tRNA (PDB 8CAM). **B.** WT conformation in presence of A-site and P-site tRNA (PDB 8EMM). **C.** Δ9 class 1. **D.** Δ9 class 4.

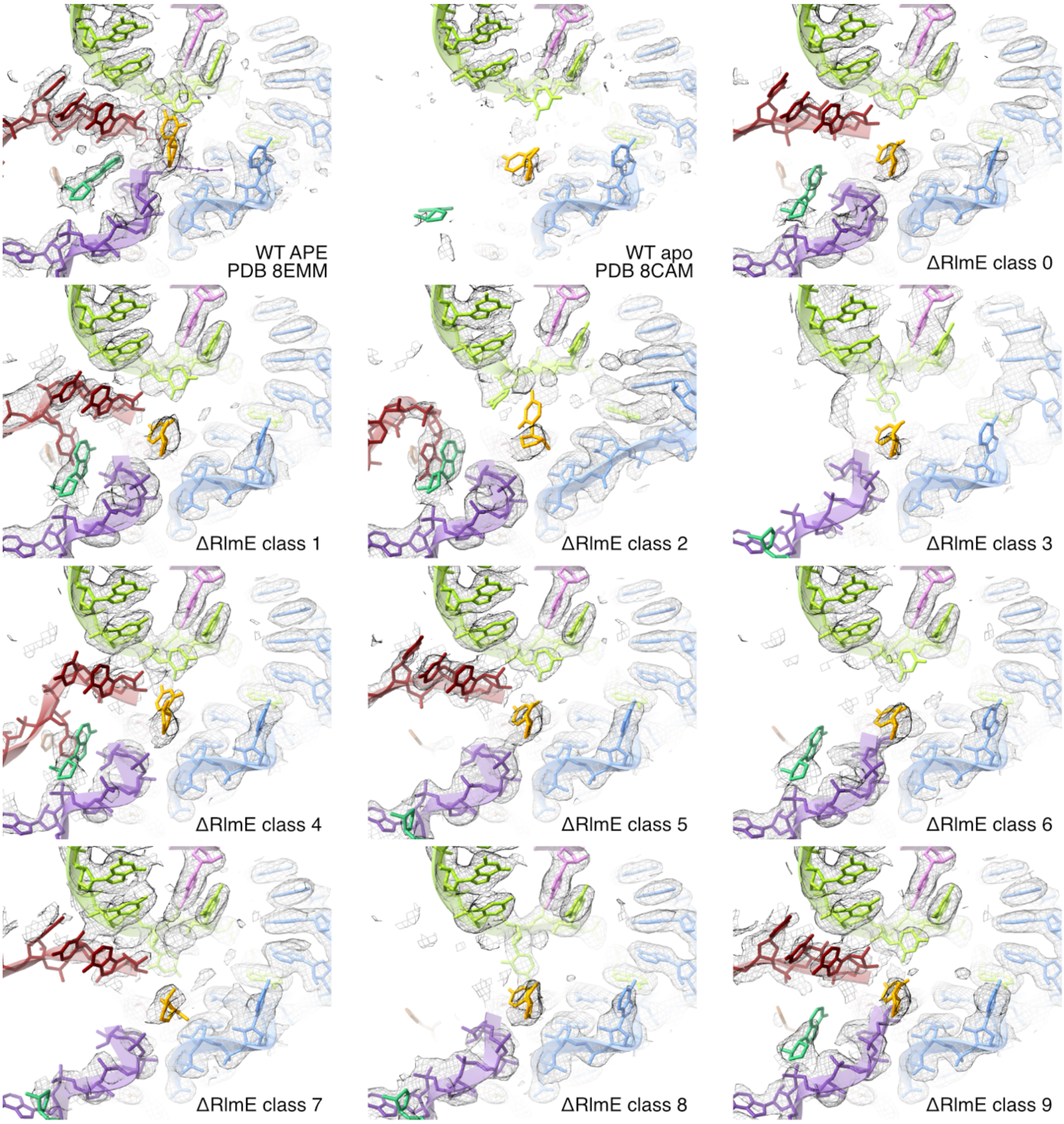

Supplementary Figure 21. Alternative stacking at the PTC in ΔRlmE classes. Same view as in Figure 7. Reference structures are WT with A- and P-site tRNA (PDB 8EMM (2)) and WT without tRNA (PDB 8CAM (5)). Colours as follows PTCα (blue) PTCγ (lime green), U2585 (orange), A2602 (mint green), G2576 (pink), A-site tRNA (dark red), P-site tRNA (purple). Segmented maps shown as mesh within 2 Å of residues in any of the classes.

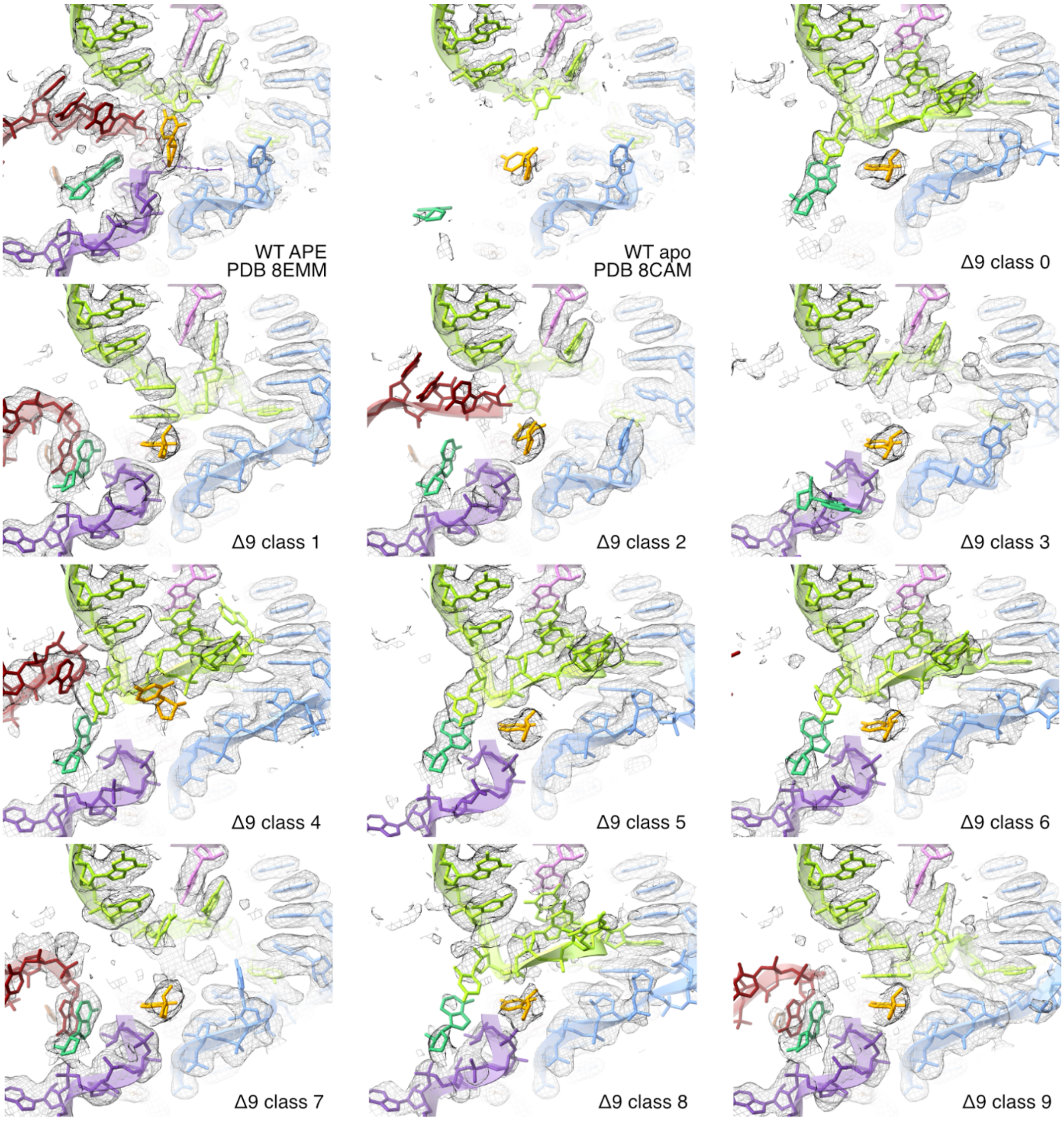

Supplementary Figure 22. Alternative stacking at the PTC in Δ9 classes. Same view as in Figure 7. Reference structures are WT with A- and P-site tRNA (PDB 8EMM (2)) and WT without tRNA (PDB 8CAM (5)). Colours as follows PTCα (blue) PTCγ (lime green), U2585 (orange), A2602 (mint green), G2576 (pink), A-site tRNA (dark red), P-site tRNA (purple). Segmented maps shown as mesh within 2 Å of residues in any of the classes.

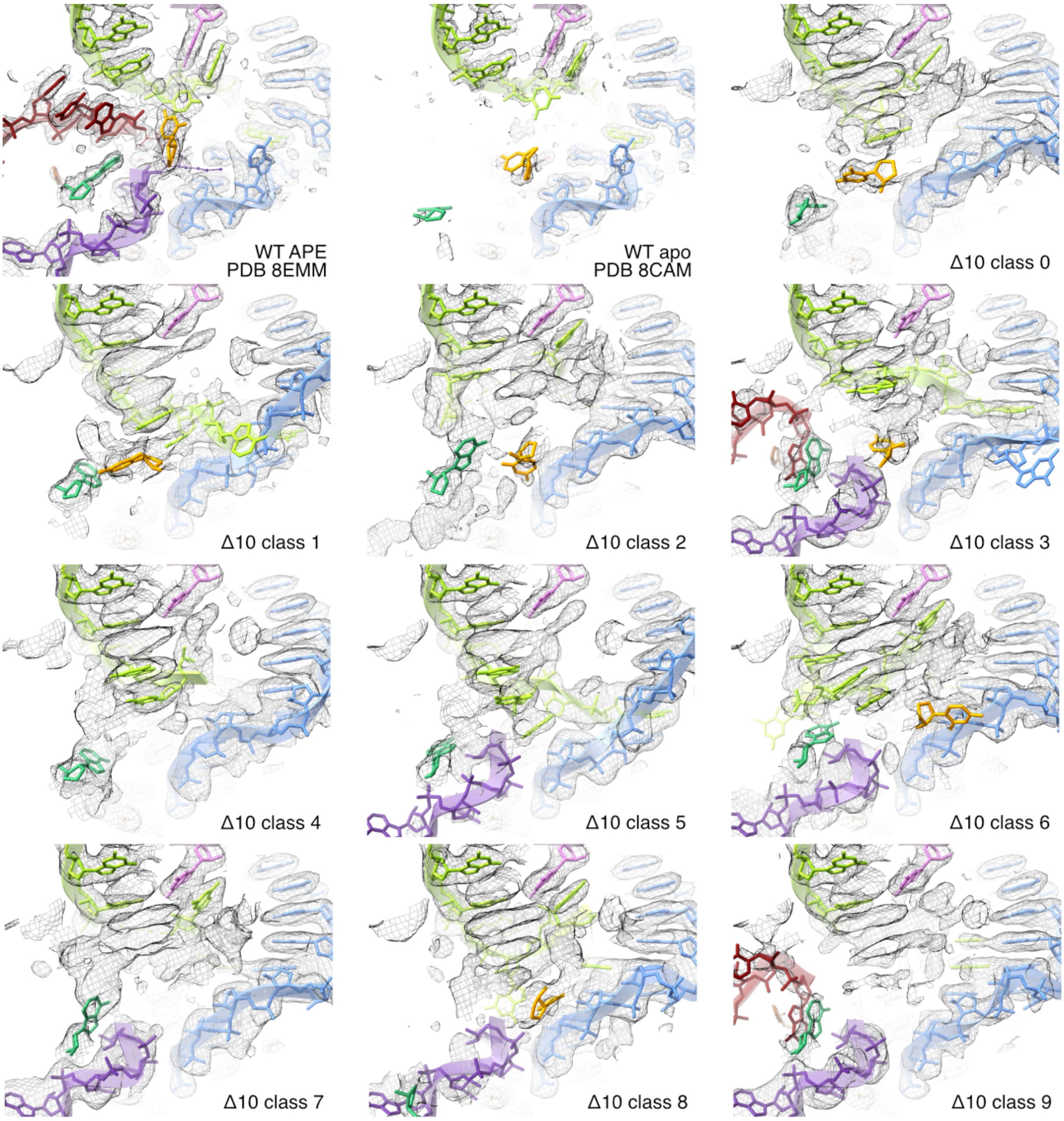

Supplementary Figure 23. Alternative stacking at the PTC in Δ10 classes. Same view as in Figure 7. Reference structures are WT with A- and P-site tRNA (PDB 8EMM (2)) and WT without tRNA (PDB 8CAM (5)). Colours as follows PTCα (blue) PTCγ (lime green), U2585 (orange), A2602 (mint green), G2576 (pink), A-site tRNA (dark red), P-site tRNA (purple). Segmented maps shown as mesh within 2 Å of residues in any of the classes.

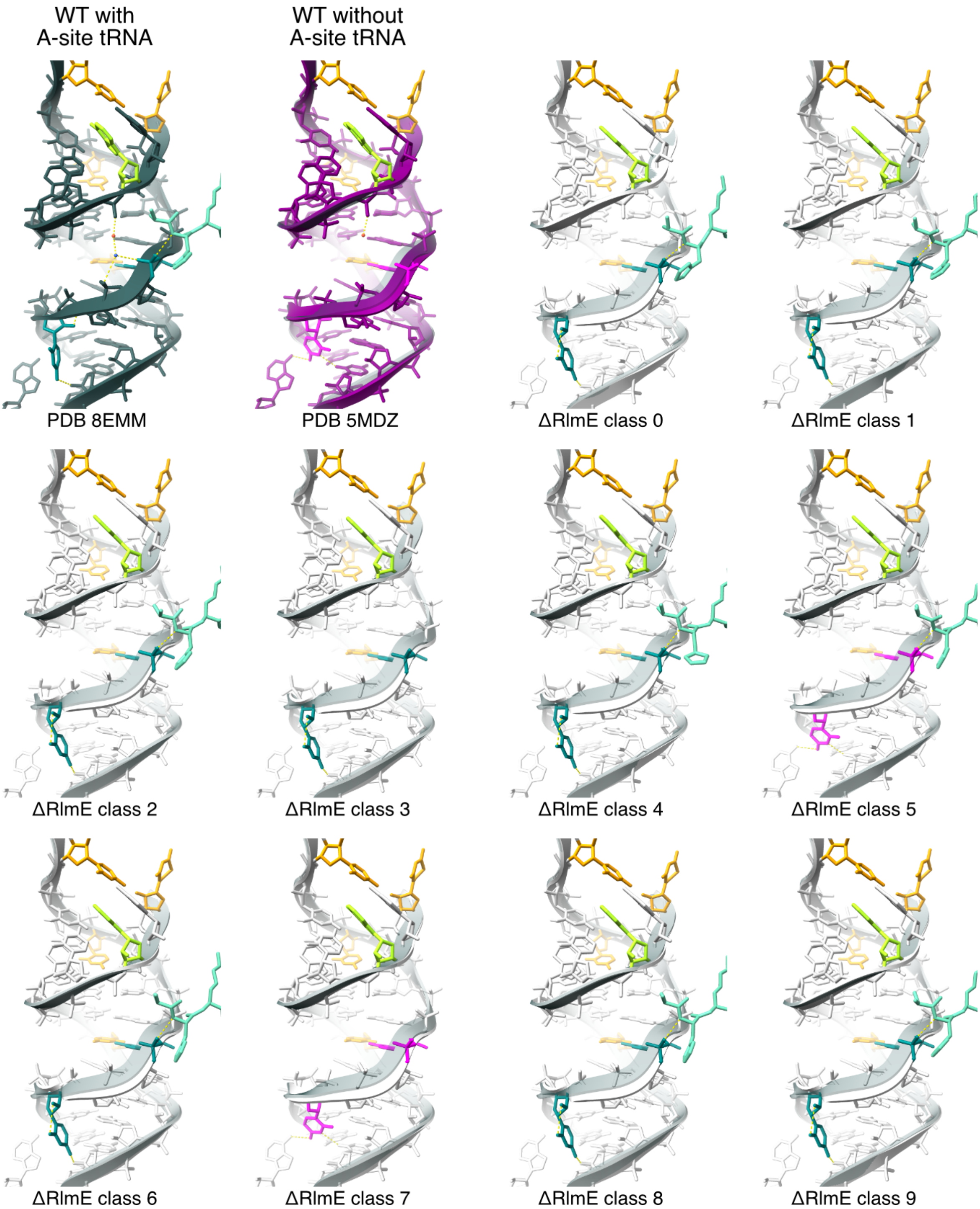

Supplementary Figure 24. Switching of U2491 and deformation of H89 in ΔRlmE classes. The view and colours are the same as in Figure 8. Reference structures are WT with A- and P-site tRNA (PDB 8EMM (2)) and WT without A-site tRNA (PDB 5MDZ (6)). U2491 in the OUT conformation is shown in teal and in the IN conformation in magenta. N-terminus of bL27 show in mint green.

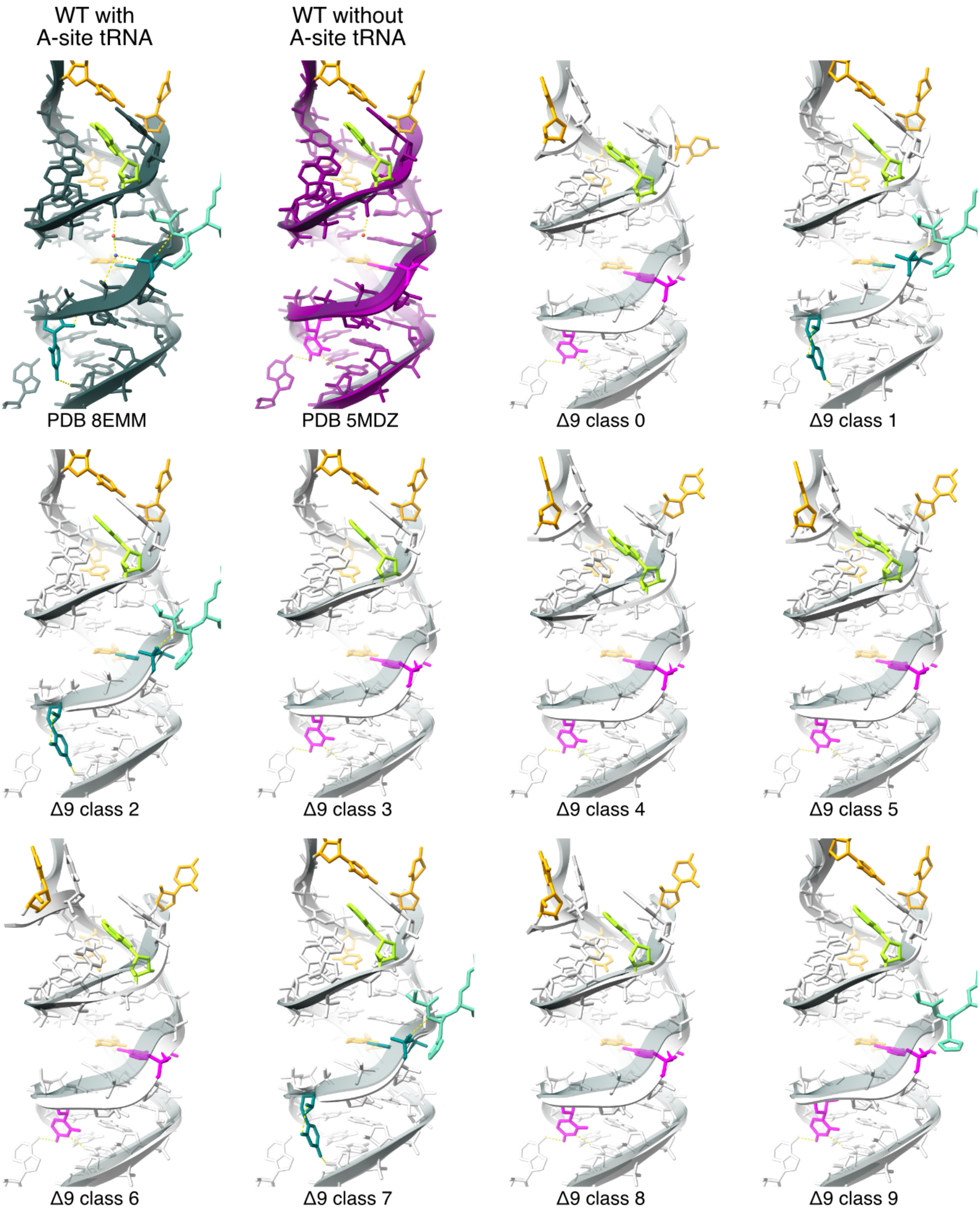

Supplementary Figure 25. Switching of U2491 and deformation of H89 in Δ9 classes. The view and colours are the same as in Figure 8. Reference structures are WT with A- and P-site tRNA (PDB 8EMM (2)) and WT without A-site tRNA (PDB 5MDZ (6)). U2491 in the OUT conformation is shown in teal and in the IN conformation in magenta. N-terminus of bL27 show in mint green.

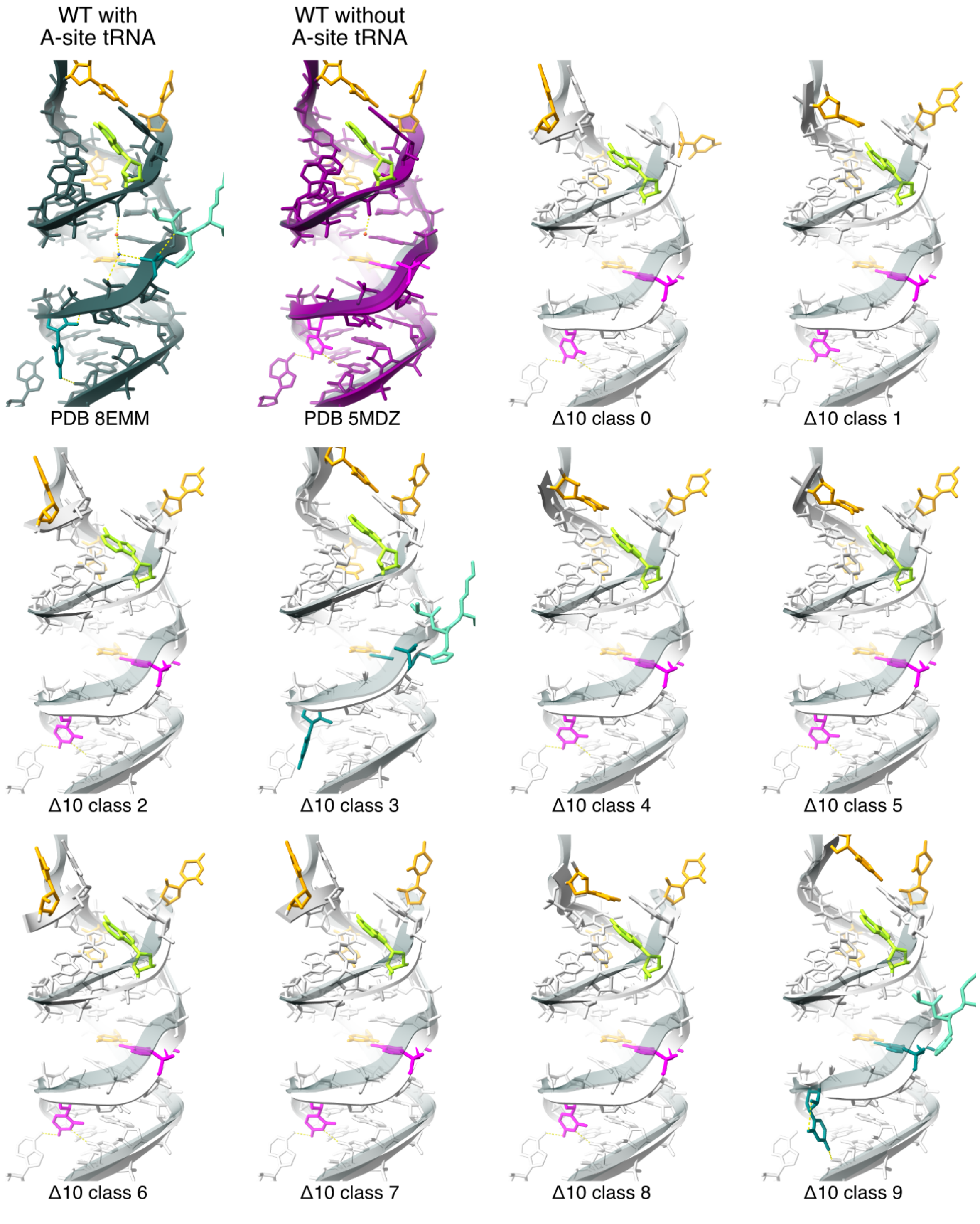

Supplementary Figure 26. Switching of U2491 and deformation of H89 in Δ10 classes. The view and colours are the same as in Figure 8. Reference structures are WT with A- and P-site tRNA (PDB 8EMM (2)) and WT without A-site tRNA (PDB 5MDZ (6)). U2491 in the OUT conformation is shown in teal and in the IN conformation in magenta. N-terminus of bL27 show in mint green.

Supplementary references

1. Petrov,A.S., Bernier,C.R., Hershkovits,E., Xue,Y., Waterbury,C.C., Hsiao,C., Stepanov,V.G., Gaucher,E.A., Grover,M.A., Harvey,S.C., *et al.* (2013) Secondary structure and domain architecture of the 23S and 5S rRNAs. *Nucleic Acids Res.*, **41**, 7522–7535.

2. Watson,Z.L., Knudson,I.J., Ward,F.R., Miller,S.J., Cate,J.H.D., Schepartz,A. and Abramyan,A.M. (2023) Atomistic simulations of the Escherichia coli ribosome provide selection criteria for translationally active substrates. *Nat. Chem.*, **15**, 913–921.

3. Forsberg,B.O., Shah,P.N.M. and Burt,A. (2023) A robust normalized local filter to estimate compositional heterogeneity directly from cryo-EM maps. *Nat. Commun.*, **14**, 5802.

4. Wang,W., Li,W., Ge,X., Yan,K., Mandava,C.S., Sanyal,S. and Gao,N. (2020) Loss of a single methylation in 23S rRNA delays 50S assembly at multiple late stages and impairs translation initiation and elongation. *Proc. Natl. Acad. Sci. U. S. A.*, **117**, 15609–15619.

5. Paternoga,H., Crowe-McAuliffe,C., Bock,L. V., Koller,T.O., Morici,M., Beckert,B., Myasnikov,A.G., Grubmüller,H., Nováček,J. and Wilson,D.N. (2023) Structural conservation of antibiotic interaction with ribosomes. *Nat. Struct. Mol. Biol.*, **30**, 1380–1392.

6. James,N.R., Brown,A., Gordiyenko,Y. and Ramakrishnan,V. (2016) Translational termination without a stop codon. *Science*, **354**, 1437–1440.
